# Mixed feelings: Unintended consequences of HIV outreach care in Zimbabwe

**DOI:** 10.1371/journal.pone.0354747

**Published:** 2026-07-31

**Authors:** Noa Gluskinos, Caroline Maposhere, Ushehwedu Kufakurinani, Godfrey Hove, Kathy McCarty, David Katzenstein, Anat Rosenthal

**Affiliations:** 1 Faculty of Welfare and Health sciences, Department of Social work, Haifa University, Haifa, Israel; 2 Biomedical Research and Training Institute (BRTI), Harare, Zimbabwe; 3 School of Global Studies, University of Sussex, Falmer, Brighton, United Kingdom; 4 Department of Anthropology and Development Studies, University of Johannesburg, Johannesburg, South Africa; 5 Department of Historical Studies, National University of Lesotho Roma, Roma, Lesotho; 6 Department of History, The University of Stellenbosch, Stellenbosch, South Africa; 7 Chidamoyo Christian Hospital, Karoi, Zimbabwe; 8 Department of Molecular Biology, Biomedical Research and Training Institute, Harare, Zimbabwe; 9 Department of Medicine, School of Medicine, Stanford University, Stanford, California, United States of America; 10 Department of Health Policy and Management, School of Public Health, Faculty of Health Sciences, Ben-Gurion University of the Negev, Beer Sheva, Israel; World Health Organization, SWITZERLAND

## Abstract

**Background:**

Decentralization of antiretroviral therapy (ART) brought treatment to millions of people in Sub-Saharan Africa. However, the implications of such decentralization are still not fully understood.

**Methods:**

The study included 60 semi-structured interviews with community leaders, healthcare providers, patients, and community members served by a rural mission hospital in Zimbabwe, conducted in January-March 2019. Interviews were transcribed and translated into English and coded using an iterative codebook. Coded extracts were grouped into themes and categories, systematically using QDA Miner qualitative analysis software.

**Findings:**

The study reveals that treatment-for-all policies and decentralization of ART have indeed improved access to care (as experienced by the study participants). However, the study shows that these policies also had unintended negative consequences. Stigma and complex social relations significantly affect the practices and experiences of ART outreach care, for both patients and healthcare providers, and may hamper attempts to reduce stigma as well as increase physical and social barriers to accessing ART.

**Conclusions:**

The study’s results highlight the need to understand the consequences of the decentralization of HIV care, as well as the barriers and facilitators associated with ART outreach in order to improve quality of care and provide true accessibility to HIV care for all.

## Introduction

The broad introduction of antiretroviral therapy (ART) in low-income countries brought urgently needed treatment to millions of people, especially in Sub-Saharan Africa. No less important, efforts to achieve the 90-90-90 targets (defined as having 90% of people living with human immunodeficiency virus/HIV knowing their status; 90% of those aware of their HIV-positive status receiving treatment; and 90% of those receiving treatment achieving suppressed viral loads) [1], and later amended to 95-95-95 targets, required focused efforts to reach an ever growing number of people. One strategy to expand access to HIV care was decentralization—shifting services from central hospitals to local clinics. The aim of this approach was to reduce barriers by providing ART closer to patients’ homes [2].

It was assumed that the introduction of the “Treat All” policy (i.e., immediate treatment of all people living with HIV regardless of their CD4 + cell count) in a decentralized system would remove existing barriers to care, reduce HIV-related stigma, and increase knowledge about HIV and ART so that patients could benefit from ART’s widespread accessibility [3,4]. However, studies have shown that these goals were not necessarily met [5,6]. Therefore, questions about the effects of the “Treat All” policy, and the extent to which it has indeed improved knowledge about ART, reduced stigma, and expanded access to care, remain to some extent unanswered. Using semi-structured interviews with community members and healthcare workers in rural Zimbabwe, we examined the effect of the decentralization of HIV care and the impact of outreach programs on patients and providers. Based on the findings of the study, we argue that treatment-for-all policies and the decentralization of ART have indeed improved access to care (as experienced by the study participants), but that they have also had unintended negative consequences that could hamper attempts to reduce stigma as well as increase physical and social barriers to accessing ART. Understanding these consequences, and the barriers and facilitators to care that are associated with ART outreach, is an important step toward improving quality of and access to care.

### History of ART and ART decentralization in Zimbabwe

In Zimbabwe, the first case of HIV was diagnosed in 1985 through blood transfusion services [7]. In the early nineties, very few people living with HIV (PLHIV) knew their HIV status. People got tested for HIV through blood donations, life insurance medical examinations, research projects, or pediatric acquired immunodeficiency syndrome (AIDS) services [8]. With no HIV treatment available, those who tested positive were encouraged to adopt a “positive living” approach and take immune boosters following post-test counseling. The period from 1995 to 2005 was characterized by a massive death-rate due to AIDS-related ailments [9]; an HIV diagnosis was tantamount to a death sentence. In the Southern African region, HIV is largely transmitted through sexual activity (primarily heterosexual), which only reinforces the stigma associated with testing, as sex and death are considered sacred subjects in the regional cultures [10].

Until the mid-1990s, there was little discussion about HIV treatment in Zimbabwe. Treatment was available only in the private sector through selected pharmacies that imported ART medications on a private basis. To mobilize resources in order to procure these medications for a national program, Zimbabwe imposed on all workers an AIDS levy, dubbed the National AIDS Trust Fund (NATF). In 1999, the National AIDS Council (NAC) was formed through an Act of Parliament with the mandate to manage the collected AIDS levy and to coordinate responses to the epidemic [11,12]. In 2002, Zimbabwe declared HIV and AIDS a national disaster, but lacked sufficient resources to translate this declaration into action until the intervention of the Global Fund (Round 5) and the United States President’s Emergency Plan for AIDS Relief (PEPFAR). Through these initiatives, the Zimbabwean government was able to start decentralizing the provision of ART to district and mission hospitals across the country. Testing for HIV still carried stigma, yet treatment could only be accessed through testing. The result was a massive wave of campaigns for HIV testing as well as for treatment literacy [13].

Following the “3 by 5” initiative, launched by UNAIDS and the World Health Organization (WHO) in 2003 to provide three million people living with HIV in low and middle-income countries with life-prolonging ART by the end of 2005 [14], the Zimbabwean government set a national treatment target of 55,000 patients by the end of 2005. The number of centers providing ART increased progressively, from five in June 2004–48 in September 2005, covering 32 districts [13]. Treatment was also provided through urban-based operational research projects such as the Development of Antiretroviral Therapy in Africa (DART) and the Zimbabwe AIDS Prevention Program (ZAPP). Rural faith-based organizations started providing treatment through the Zimbabwe Association of Church Hospitals (ZACH) through the United States Agency for International Development (USAID) and the Global Fund [13,15].

Civil society organizations collaborated with the Zimbabwe National Network of People Living with HIV and AIDS (ZNNP+) to reduce stigma and increase community access to HIV prevention and treatment services through the formation of community support groups and ART outreach sites [16]. A stigma index study was conducted by ZNNP+ in 2014 to assess levels of HIV-related stigma. According to that report, more than half of the respondents experienced some form of stigma and discrimination (gossip, verbal insults, social exclusion from functions, expulsion either from work or place of residence, and physical attacks or threats). It was also noted that in some PLHIV, the internal stigma, negative beliefs, thoughts, and behaviors toward oneself, could cause emotional distress and lead to depression, and these factors were also a significant barrier to care [17,18]. Adolescents and young adults living with HIV in Zimbabwe reported HIV-related self-stigma either occasionally or frequently, and this report revealed that it was likely to be felt particularly by marginalized populations living with HIV [19]. Internal stigma was reported to be significantly reduced through individual and group interventions [20] and through a range of cognitive-based, inquiry-based, creativity-based, and mindful-based techniques, often with a forward-looking goal-setting focus [17,18,21]. Given the history of ART in Zimbabwe, efforts to expand access and fight stigma are at the heart of the decentralization of care, and thus should be understood as they unfold over time.

### ART knowledge and stigma

Studies on HIV care and stigma have yielded mixed results when it comes to the effect of the “Treat All” policies on the reduction of HIV-related stigma. Based on a survey conducted in rural Tanzania, Agnarson et al. (2013) showed that these policies did not reduce stigma, nor did they increase knowledge. Stigma was strongly grounded in lack of knowledge, mainly due to lack of formal education [22]. A United Nations General Assembly Special Session on HIV/AIDS (UNGASS) included HIV knowledge among people ages 15–24 as one of its core indicators for tracking national HIV programs [3]. Based on the data from the UNGASS questionnaire, Chan and Tsai (2018) concluded that there were no substantive improvements in knowledge about HIV in the ART era. Their findings suggest that decades of efforts at the national and international level have been largely ineffective in improving HIV knowledge [3]. Moreover, the efforts made by non-governmental organizations (NGOs), investing scarce resources in the promotion of treatment literacy and emphasizing the responsibility of individual patients as a public health strategy, have met with limited success [4]. There is a consensus in the literature that the expansion of ART alone will not result in stigma reduction. According to Maughan-Brown, in South Africa, even in the years of massive treatment expansion, stigma reduction did not occur [23]. Some studies have argued that the paradox of increasing stigma during times of increasing treatment can be explained by the social desirability bias influencing respondents’ answers [24,25].

However, other studies have shown that ART does contribute to the reduction of stigma, as patients regain their physical health and return to normal life. These new patterns of illness management have linked receiving ART to renewed social acceptance and support for patients [26,27]. Studies have also shown that ART reduces stigma through the mechanism of appearance, as patients in treatment can no longer be easily identified as sick. Thus, ART has changed the perception of HIV as being linked to death [28]. The broad introduction of ART also encouraged the establishment of support groups of HIV positive and negative individuals. A study conducted in Zimbabwe showed that being part of a support group reduced participants’ perceived stigmatization levels [29]. Support groups for HIV-positive individuals, mainly those hosted in ART clinics, have also been shown to reduce internal stigma [28]. Thus, two conflicting trends have been identified in the scholarship: ART reducing HIV-related stigma on the one hand, and ART increasing such stigma on the other [30]. These conflicting findings point to a complicated reality in which HIV-related stigma remains an issue that requires further investigation.

Stigma is understood as a barrier to care at all stages, from testing [31] to initiating ART treatment [32,33] to ART adherence and follow-up [34–37]. Studies have also explored the mechanisms through which stigma affects patients’ illness management, and have shown that stigma has an impact on mental health and illness disclosure, and may lead patients to experience difficulties in initiation and adhering to care [38,39]. Studies in Ethiopia and Nigeria showed that stigma could also be expressed by healthcare providers through acts of impoliteness, fear of touching and handling HIV patients, financial discrimination, and breaching of confidentiality [40,41]. Moreover, studies in Malawi, Botswana, and Namibia have shown that healthcare providers’ stigmatization of patients worsens where sexual minorities and other marginalized groups are concerned [42–45].

In situations in which ART is accessible and available, HIV can become a manageable, chronic condition rather than an acute, life-threatening illness. Studies have maintained that ART may act to normalize HIV and create states of “chronic normalcy” [46,47]. However, for many PLHIV, chronic normalcy is far from the reality. A study conducted in Tanzania found that ART created a certain normalization through reducing internal stigma, but it did not reduce the communities’ discriminative attitude toward PLHIV [48]. In the context of extreme poverty, normalcy cannot be achieved, given challenges of food scarcity, lack of permanent income, and lack of drug supplies [49]. In low-resource environments, there is a gap between the institutional approach, which advocates for normalcy, and the lives of PLHIV, which are characterized by ongoing uncertainty [47].

In such uncertain, resource-limited environments, ART has had unintended consequences. In Mozambique, Kalofanos (2010) documented the impact of hunger, conflicts related to food support, and ART [50]. As PLHIV now live longer, more food is needed for them; in other words, their medical coverage has improved, but their life conditions have not. Therefore, food donations directed only toward PLHIV have also created conflicts and tensions among communities. Other studies have shown the unintended consequences of stigma itself, framing it as a pull-push factor to care, and not as a barrier [26,51]. These studies have shown how ART can help disguise sickness status, by making patients un-symptomatic, and therefore help avoid stigma. In this way stigma is the “pull factor” for adherence (20, 45). In terms of stigma being a “push factor,” ART is given in community treatment centers (CTCs) or voluntary counseling and testing centers (VCTs), making patients more visible and separated from other health services, and therefore more exposed to stigma [5,6]. The literature on HIV-related stigma reveals a complex reality, where the relationship between increasing treatment and knowledge does not necessarily lead to stigma reduction and thus causes various forms of unintended consequences. These consequences are often a barrier to care.

## Materials and methods

### Study setting

The mission hospital at the heart of this study is a hundred-bed facility established in 1968, and is a member of the Zimbabwe Association of Church Hospitals (ZACH). The hospital officially serves a rural catchment area of 32,000 people, but the actual patient population extends beyond its locality, as patients from nearby communities are often served in its facilities. The hospital started testing for HIV in 1985, although no treatment was offered at the time. Prevention of Mother to Child Transmission services started in 2000 (Single-Dose Nevirapine, the Southern African standard of care at the time). The hospital’s HIV clinic started providing care to PLHIV in 2002, initially on the basis of disease stage. Since 2017, the hospital has offered care for all HIV-positive patients under “test and treat” recommendations. A pediatric HIV clinic was established in 2004. Acknowledging prevailing barriers to care, the mission hospital established eight HIV outreach sites in 2012. At the onset of this study in 2018, hospital HIV services reached a population of 2418 patients, of whom 1464 received care on hospital grounds, whereas 954 received care at one of the outreach sites.

### Study design

This article was drawn from the ethnographic arm of the CBART (Community Based ART) studies, a collaboration of community-based studies conducted in Zimbabwe from 2018 to 2020. The CBART ethnographic study was conducted independently and designed as a multi-site ethnography [52] that followed the work of community-based ART clinics at the outreach sites served by a rural mission hospital.

### Data collection and processing

As part of the CBART ethnographic study, we conducted 60 semi-structured interviews with community leaders, healthcare providers, patients, and community members in the areas served by the mission hospital and its outreach clinics. Participants were recruited using purposeful snowball sampling to include a variety of perspectives and experiences. The interviews included questions about ART services in Zimbabwe, the work of the mobile ART clinics, the availability of services, and the impact of community-based ART on the interviewees’ communities. The interviews were open-ended to allow for a variety of answers, and to evoke detailed respondent narratives.

Participant recruitment started on January 13, 2019 and ended on March 1, 2019. The study team worked with community health workers to introduce the study to community members and invite potential participants. Following the broad invitation to join the study, shared via community health workers and community leaders, interested participants were introduced to the study team. The interviews were held in participants’ homes or other local settings of the participants’ choosing. The interviews were conducted in Shona by native Shona-speakers and transcribed and translated into English.

### Data analysis

**Fig 1:**
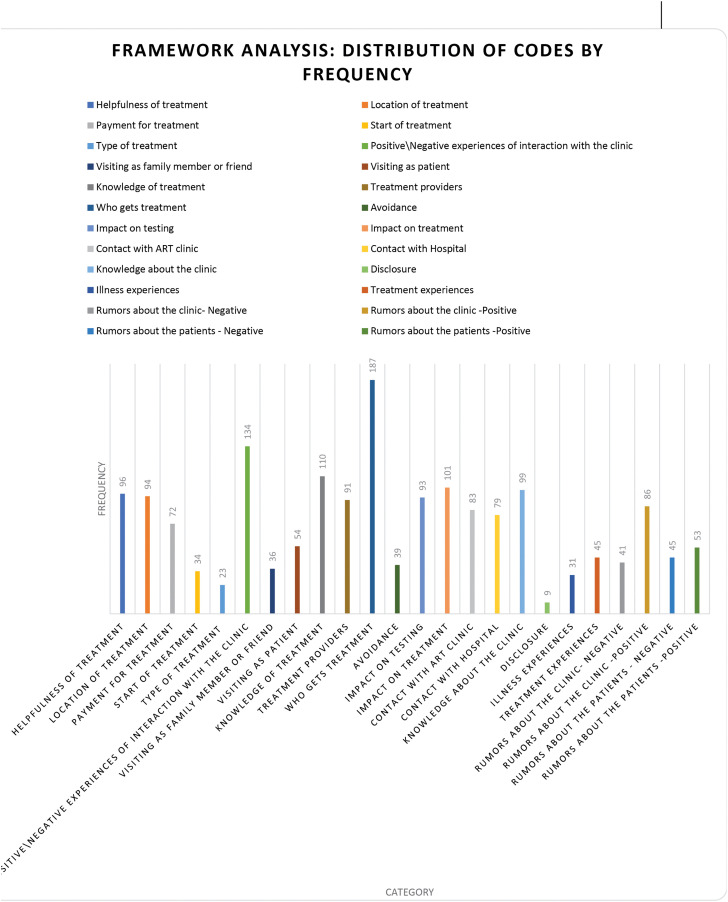
Framework Analysis – Distribution of Code by Frequency.

All interview translations were conducted by teams of Shona-speaking researchers, in consultation with Shona language experts to ensure maximum accuracy. In the preliminary round of coding, members of the study team read the transcripts, and a transcript sample was coded using a preliminary codebook. Native Shona speakers who conducted the interviews took part in the preliminary coding phase to track inaccuracies in translation. Following the preliminary round of coding, a smaller section of the team revised the preliminary codebook based on feedback from the preliminary round and simultaneously coded sample interview transcriptions to resolve discordant codes. The updated codebook was used to code all transcripts and included 24 codes (Fig 1). We compared the codes within and across transcripts and summarized coded extracts grouped into themes and categories systematically using QDA Miner qualitative analysis software. Drawing from Braun and Clarke [53,54], the themes and categories were used to lead the thematic analysis grouping them into a narrative highlighting the study’s results.

### Ethical considerations

All participants provided written consent and signed a consent form, witnessed by the interviewer and community health worker. All participants were above 18 years of age. To protect the privacy of potential participants, the team did not approach individuals directly. Ethical approvals were gained from the Biomedical Research and Training Institute (AP143/2018) and Medical Research Council of Zimbabwe (MRCZ/A/2329). No payments were made to participants. However, in consultation with the mission hospital and in accordance with existing practices, participants received a token gift (a bar of soap) for their time and effort.

## Results

Sixty individuals from the communities served by the mission hospital were interviewed. The sample included 34 (56.67%) women and 26 (43.33%) men, between the ages of 24–71. The majority of participants reported having a high school education or higher (n = 41; 68.33%). Of the interviewees, 7 (11.67%) were healthcare workers, 9 (15%) were community leaders, 7 (11.67%) were patients of the ART clinics, and 37 (61.67%) were community members. Finally, although not required to respond to the question, 29 participants (48.33%) reported their HIV status to be positive, and 30 (50%) participants reported their HIV status to be negative. One participant did not disclose their status (see Table 1).

**Table 1:** Interview Participant Characteristics.

Characteristic	Frequency (n)	Percentage (%)
Gender		
Male	26	43.33%
Female	34	56.67%
Age		
Age – Median (Range) 44 (24–71)		
HIV Status		
Positive	29	48.33%
Negative	30	50.00%
Unknown	1	1.67%
Education Level		
No formal education	2	3.33%
Primary	17	28.33%
Secondary	32	53.33%
Post-Secondary	9	15.01%
Marital Status		
Single	4	6.67%
Married	40	66.67%
Divorced	4	6.67%
Widowed	12	19.99%
Number of Children		
0	5	8.33%
1-3	27	45.00%
4-6	25	41.67%
7+	3	5.00%
Served by Mission Hospital and Clinics		
Yes	47	78.33%
No	13	21.67%
Interviewee Group		
Healthcare providers	7	11.67%
Community leaders	9	15.00%
Patients	7	11.67%
Community members	37	61.66%

*The median and range for participant age are presented excluding one instance where age data was not available (recorded as “0”). This exclusion was implemented to provide a more representative measure of the central tendency and dispersion of the known ages within the study sample.

**Fig 2:**
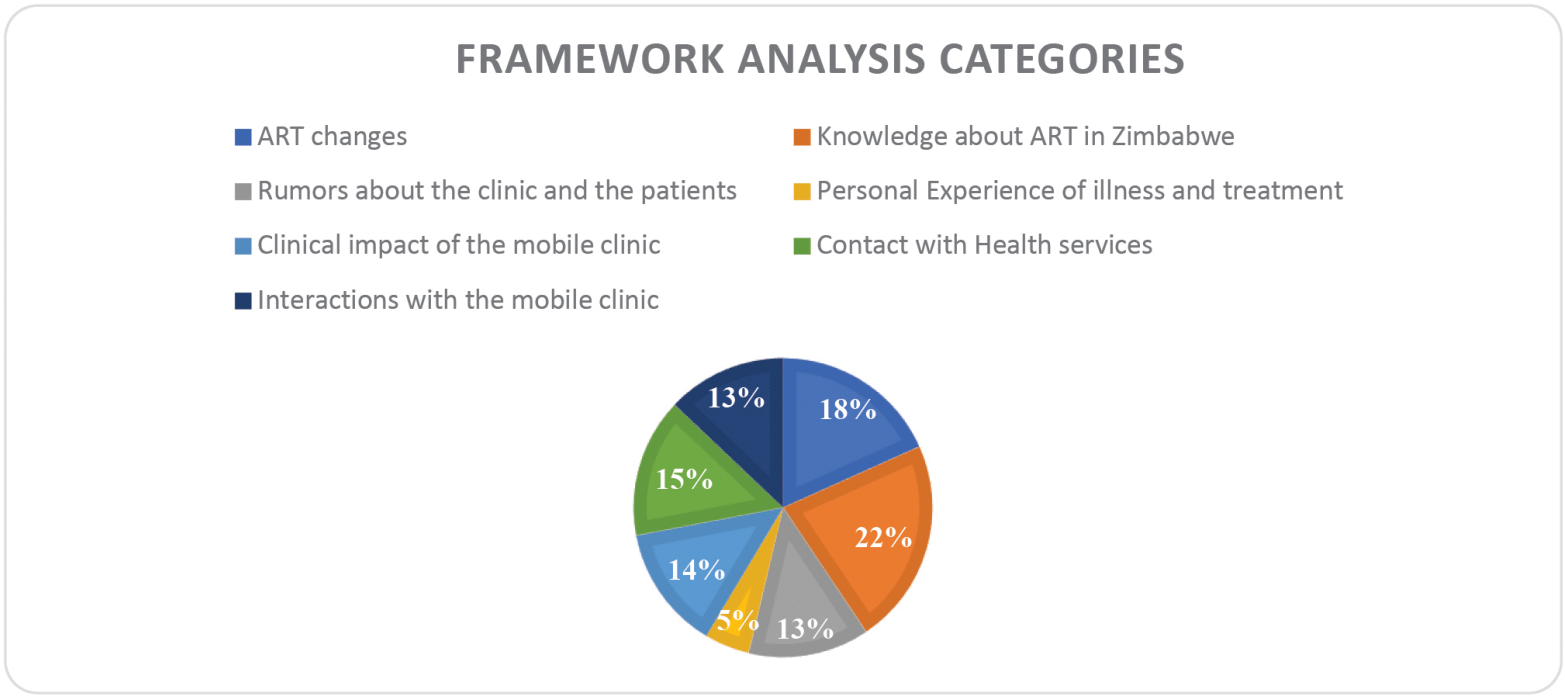
Framework Analysis Categories.

Seven categories emerged from the coding and analysis of the interviews: knowledge of ART in Zimbabwe; changes to ART programming; participants’ contact with health services; types of interactions with the mobile clinics; the mobile clinics’ impact; personal experiences of illness and treatment; and rumors about the clinics and the patients (Fig 2). Based on these categories, we conducted a narrative analysis of the data [53,54] to explore the impact of the decentralization of care and the introduction of ART outreach clinics, highlighting knowledge of ART, stigma, and the experience of navigating care. The quotes presented in the results section are representative of the overall data set and highlight the major themes and trends stemming from the interviews.

The expansion of ART to all PLHIV as a result of the 2015 WHO guidelines [55], and the adoption of the 90-90-90 targets in Zimbabwe, radically changed the landscape of care for Zimbabweans. No less important, the adoption of outreach strategies provided an opportunity for real change. Provided in close proximity to patients’ home, advertised in advance, and dramatically reducing the physical barriers to care that resulted from hospital-based services and the long-lasting stigma that resulted from treating only the gravely ill who came to the hospital, ART outreach services presented an opportunity to change the face of ART and address its persistent shortfalls.

However, although ART outreach care carried the promise of change, the reality of receiving care in rural communities brought new challenges for patients and providers, some of them unexpected. Thus, alongside the growing access to services and knowledge, old fears of stigma and new challenges of navigating landscapes of care shaped patients’ ART experiences.

### “Their perception is now different”: Knowledge of ART in the age of outreach

The broad introduction of ART in rural communities in Zimbabwe included a massive information campaign. Following decades of HIV/AIDS education in the Sub-Saharan African media, which yielded mixed results at best (2), the expansion of ART, and the broad reach of outreach clinics, included more information on prevention and treatment.

No longer limited to the confines of a hospital or a clinic, HIV care was now administered in a designated area of the market, or a school in the village. Receiving care required patients to assemble at a previously arranged time and place, along with other patients from the community, and become educated about their disease together. Addressing these experiences (i.e., of gaining more knowledge), a man who was living with HIV said in an interview:

People are getting great help because in the past years we lost many people because of lack of knowledge. But, because people continue to share knowledge, especially in this community, and if someone tells me that my friend is not feeling well, as a friend it won’t be hard for me to approach them with advice, and in some cases, people survive if they listen. (Case 24/Interviewee F03)

The relation between outreach care and knowledge was also addressed by participants who were aware of the benefits and complexities of such care. In the words of another participant, a male farmer who was HIV positive:

People did not really understand much about HIV/AIDS. But with the education we have been getting though the outreach programs and how they treat people, one would not be able to tell that a person is HIV positive. Many who did not want to come before, now visit the clinic because they see the benefit it is bringing… the numbers are growing, but there are still those who are not knowledgeable, who are stubborn, but the percentage of people informed about HIV is growing. (Case 50/Interviewee A14)

This new knowledge regarding care options, as well as the changing landscape of care, meant that being, and looking like, a person living with HIV went beyond mere knowledge of the disease and treatment options. A shift had been created in how an HIV diagnosis was experienced and understood. A local female farmer who was living with HIV explained:

Yes, it is different [now] because long back, people were afraid. If someone was diagnosed with HIV, the person would get so stressed to the extent that some would decide to take poison, feeling it is better to kill themself because they are HIV positive. That person would not want those they stay with or the community to know that they are positive.

Q: Why is this so?

A: Due to fear of being laughed at and not being treated well and being discriminated against for being a sick person.

Q: So, have the people’s perceptions on HIV in your community changed?

A: Yes, their perceptions are now different. Now we are all just staying together the same way and well, those with HIV and those who do not have it. People now see that we are just one, there is no discrimination. Way back, people would really discriminate each other, and when people would know you are on ART, they would laugh at you and word about your status would go around the whole community, but it is now better. (Case 36/Interviewee E05)

Similar sentiments addressing the ties between the available options of care, and the perceptions associated with an HIV diagnosis, were expressed by other interviewees. One of the village headmen said in an interview:

I think people have better and improved understanding. This is because if we look at the time when treatment was introduced people were ashamed and embarrassed to go [and] collect [drugs] because there was some humiliation associated with being known to be HIV positive. People did not understand the disease (HIV) and they were afraid of the associated stigma, but now people are open to even describe how the medication has been improving their health. So, I think people now understand. (case 1/interviewee D03)

This additional knowledge, and the introduction of care, have acted to reduce stigma. The reduced stigma was mentioned not only in the context of outreach care and HIV education, but also framed by participants as a process of gradual change, as a male farmer who was HIV positive explained:

Initially there was discrimination, they feared handshakes and sharing stuff such as food, cups and body contact with people with HIV. It was basically due to lack of knowledge and ignorance. … [Now] even stigmatization is lessening as they get to know one can be born with it [HIV]. So we are even getting back to playing together and socializing (case 49/interviewee A13)

No less important, the availability of ART in remote rural communities allowed an extrapolation from HIV to other diseases, one that could even be seen as a form of normalization [47]. As one female farmer who was HIV positive said:

Nowadays people no longer discriminate. They see us like any other individual, people now just see someone with HIV and someone with malaria as just the same because the virus is not spread through touching someone’s skin or helping someone. It is a virus which is inside the blood, so you cannot just spread it like that. So, people have come to realize that we are just the same. (case 36/ interviewee E05).

Although this sense of “sameness” and normalcy was shared by many interviewees, and accords with findings from similar studies in the field [3,4], a deeper analysis of participants’ experiences revealed a more complex picture of HIV, rumors, and stigma in the participants’ communities.

### “Speaking the words of ridicule”: The persistence of HIV stigma in outreach care

The expansion of ART, and its proximity to everyday life in the community via the use of outreach sites, may have helped some patients and community members gain more knowledge about HIV care and new ideas of normalcy, as seen above. However, for many others, the introduction of ART and outreach care did not necessarily solve the problem of HIV-related stigma.

In accordance with the familiar patterns of stigma known since the 1980s and the early days of AIDS, also prevalent in Sub-Saharan African countries [56], interviewees were often quick to talk about HIV-related stigma in their communities. A widow who was living with HIV explained her understanding of HIV labeling:

Is there anyone who likes a person who is living with HIV? No one likes a person living with HIV. A person who does not have HIV actually sees as if he/she is never going to die, but there is one God and he is for everyone. We, people living with HIV, are not even seen as human beings. We are not seen as people for sure and this is true. (case 34/interviewee E03)

Addressing the need to cope and respond to the prevalent stigma, another interviewee, who was a local pastor, explained the impact of stigma as well as how it shaped the narrative of illness and infection:

It’s because some people are afraid of being looked down upon because they are ill. They fear that when people spend time doing certain things, they may be excluded in an attempt to protect their health. They do not want to appear weak; they want to maintain their positions. They will also be trying to avoid being talked about and scorned by people who will come up with stories of how they would have gotten infected. (case 29/Interviewee F08)

Alongside the familiar patterns of stigma, study participants also reflected on more subtle expressions of stigma, often phrased as humor or jokes, but still noticeable by many, as a female farmer who was HIV negative shared:

A few people still remain with the old behavior of scorning. But now people see that the people we say have HIV look healthier than us, their bodies are fit. Here and there people mock but many times they do it jokingly saying “you now look well thanks to the pills”. (Case 22/ interviewee F10).

Whether comments were made in jest, or were insults, or gossip, study participants shared their complex experiences with stigma as it sometimes changed character, alongside the changing bodies of those undergoing treatment. As seen elsewhere in the region [57], the impact of ART on those receiving treatment was often incorporated into stigma, and did not necessarily abolish it.

Experienced and internal stigma [58] not only shape illness experience but also impact access to care. Known as a significant barrier to HIV care [31,34–37], stigma, and the fear of stigma, also affected care in the outreach settings under discussion. Although outreach care was envisioned as something that would break the barriers of physical access and social unacceptability, the reality was more complex. A local teacher illustrated this aspect when describing the activities of the outreach clinic in his community:

There [is] usually a car at the shops, that’s where they get their tablets. But it is not all of them that get their tablets there because there is an issue of stigmatization. Some people therefore choose to go to the main center … So, some go to the center, but some still get their medication here. (case 3/interviewee D05)

Stigma as a barrier to care was also discussed in the interviews with healthcare workers and patients. From the healthcare providers’ perspective, stigma was still present in their interaction with patients who were invited to come for care, as one of the healthcare providers explained:

Some just do not want [to come to the clinic], we even tried to talk to them. They would rather visit the clinic when they are seriously ill, being ferried in wheelbarrows… They are frustrated and do not want people to know that they visit the clinic, or that they are HIV positive. If you go there, it becomes obvious to everyone that they are either positive or that they would have been promiscuous (Case 44/ interviewee A07).

The same sentiment was shared by patients as well, as male farmer who was HIV positive elaborated:

Even these days some are still dying because there are ashamed of people knowing their HIV status. If tested positive, an individual may possibly find it difficult to accept her or his HIV status and possibly think that it is now the end of their lives. But this is not true, it is now the opposite. It is because where we are given pills, there are a lot of people and it is an open place. Even if you go to the hospital, you will be holding cards which are different from others, meaning everyone will know your HIV status. But if you know you are positive you have to accept your situation and there should be nothing to be ashamed of or to be afraid of because what is now important is my life and to fend for my family (Case 51/ Interviewee C10).

The choice to escape stigma by avoiding the outreach sites, and electing to receive care in more remote locations, was a common practice. One male patient commented:

Currently, as I have noticed at the village mobile clinic, the people who collect medication there are the same people who have always collected from there … there are no new faces collecting at the village because people are still living in fear and shame. (case 24/interviewee F03)

Addressing the causes of stigma and its impact on the program’s acceptability in the community, he continued:

Others have a tendency to speak scornfully when they hear that someone is on ART. They say a lot of words of ridicule and that is retrogressive, holding back the program`s success. This is because other people who would have been buying the idea of opening up, end up hiding because of the words of society. What really pains me the most is that someone who will be speaking words of ridicule may have never been tested in their lives, and has no idea of their status but demeans those already on the program (case 24/ interviewee F03)

Shaping choices of how to receive care as well as illness narratives, stigma went hand in hand with people’s newfound knowledge of HIV and the new ART services provided in rural communities. This complex relationship extended to other social relations and access-related decisions, and was at the heart of the conflict between the benefits and barriers associated with outreach care.

### “Mixed feelings”: The conflict between benefits and drawbacks of outreach care

Living with HIV and receiving ART services in an outreach clinic presented community members with many challenges. In addition to the stigma, disclosure, and treatment adherence, and the extreme poverty, food insecurity, and unstable employment—all known and prevalent factors in the region [4,49,50,59–68]—contradictions stemming from their lived experiences plagued many of the interviewees. Interviewees used words such as “mixed feelings,” “embarrassment,” and “feeling troubled,” and struggled to negotiate the benefits of care with the drawbacks and barriers they encountered.

When talking about the benefits of receiving ART in outreach clinics vs. other ways of receiving ART, stigma, gossip, and rumors were the main barriers. A female community health worker explained:

When people comment about the people on ART themselves, they have mixed feelings because they are human but if we look at it closely, it’s actually better because people are alive and have good health. Initially, some found it difficult, others are shy to be known to take HIV medication but with time people get used to it, and even those who gossiped have become gradually quieter. Gossip is what makes it hard for some. But now it’s better because people are now getting used to it since we sometimes speak to them. (case 23/interviewee F02)

The difficulty of coping with gossip in the outreach settings, mentioned by many interviewees as a barrier to care, resulted in creative solutions such as traveling to clinics in remote communities where patients felt they could maintain their privacy. Although quite sensible in many ways, these tactics conflicted with the very rationale that underpinned the outreach model, and the benefits of receiving ART close to home. The local pastor explained these choices:

Yes, from mobile clinics I hear similar reports. For example, there are many people who do not collect their medication from the village outreach clinic when the mobile clinic comes because they do not want people to know that they are on ART, hence they go to the district hospital. Then there are those who have no problem with being known and they openly collect their medication at the village. (case 29/interviewee F08).

Even at the hospital, some patients were still concerned about privacy, and used various strategies to keep their HIV status secret, as the pastor explained:

Yes, I once worked at the hospital, I noticed that when other people come to collect medication at the hospital sometimes, they do not want people to know their status. So, they come hiding their [patient] cards and only bring them out when they are being served. On the other hand, others do not care and they come exposing their cards because they will be confident that no one knows them at the hospital. Most people do not want their status exposed (Case 29/ interviewee F08).

An interesting observation on the same topic was made by another interviewee, a male farmer who was HIV negative, who focused on processes of change, and what may have seemed like a change in patterns:

In earlier days people would not want to collect their medication from the outreach clinic but would secretly go to the district hospital because of the fear of what people would say about them. Now a few people stigmatize and talk negatively but most of us now know that it is better to come out openly and get treatment if you are found HIV positive. (case 28/interviewee F07)

The tensions caused by the conflict between benefits and barriers, and the fear of exposure in community outreach clinic settings, did not result in avoidance-based solutions only. The situation also allowed interviewees to imagine possible solutions to the problem. One of the clinic’s patients, a female farmer, explained:

I face a difficult time when I go to the district hospital.... They should make us, people living with HIV, with our cards go to a separate room and get treatment there. But at the hospital we will be mixed with everyone else, including those who just want medical attention for other things. This makes people living with HIV uncomfortable to go to the hospital because there are other people who laugh when they realize that you are living with HIV at the hospital. Some will ask themselves what their friends, relatives and neighbors will say if they see them receiving HIV treatment. So, I feel they should put us in a separate room where we receive attention. (case 31/interviewee E01).

The search for a solution that would protect ART patients from ridicule or finger-pointing stands in stark contradiction to the ideology behind bringing care into the community as a tool that would normalize ART [5,6]. This search also lies in contrast with the numerous studies in Sub-Saharan Africa that have addressed the stigmatizing nature of ART spatiality in landscapes of care, namely addressing the notion that the physical separation of ART care increases stigma and acts as a barrier to care [41,69–71].

Confronting the need to negotiate benefits and barriers, and to find ways to avoid the stigma associated with ART, interviewees not only addressed creative solutions such as receiving medication elsewhere or imagining the optimal landscape of care. They also talked about compromise or, as one interviewee called it, “acceptance.” Balancing HIV status, remoteness, poverty, stigma, and available outreach care, some patients knew that compromise was necessary. A widow living with HIV explained:

Maybe if they open a clinic close by, where people can go and get tested privately. Someone can just go to that clinic without anyone knowing why they are there; this might make it easier for people to actually get tested. It is the same with us people living with HIV when we started getting medication at the village outreach clinic. At first, we were only 2, they would tell us to go and sit at some spot with our cards waiting for our drugs, and everyone would just be looking at us and we would feel embarrassed and not know what to do. But we ended up accepting it because there is nothing we could do about it, and it’s your life. But with time, we ended up seeing many getting drugs there. (case 37/interviewee E06)

“Mixed feelings,” “embarrassment,” and “feeling troubled” were all part of the experience of receiving care at outreach clinics. However, these feelings were also accompanied by processes of change, negotiation, and compromise. The complexity of the experience of ART outreach care, and the need to balance benefits and barriers, shaped some of the unexpected consequences of outreach care.

## Discussion

The broad introduction of ART changed the “death sentence” associated with HIV and enabled people from low-income countries to access treatment [26–28]. This development raised high hopes that available treatment would eradicate the stigma surrounding HIV [3,4].

Since 2004, the Zimbabwean government has encouraged the decentralization of ART to districts and mission hospitals [13].The idea behind decentralization of care, broadly defined, is the relocation of services from centralized locations such as hospitals and major health facilities to lower-level health facilities such as rural and outreach clinics. The rationale behind decentralization processes was the removal of barriers to care by providing ART in close proximity to patients’ homes [2]. However, decentralization was not enough to guarantee access to ART in all rural communities. Decentralized ART, and the introduction of outreach models, was envisioned as not only a technical solution to the problem of remote communities, but also as an opportunity to alleviate the social burden of HIV. Nevertheless, although these policies helped save many lives, studies from across Sub-Sharan Africa have shown that they have not been free of controversy [24,64,69].

As shown by the results of this study, the decentralization of ART services in the studied communities indeed brought with it more knowledge of HIV, as well as opportunities to treat it. This decentralization also, in some cases, created a stronger sense of “normalization” surrounding HIV, allowing this disease to be viewed as just another disease, echoing notions of chronic normalcy observed in other areas of the world [46,47]. However, this new knowledge has not necessarily provided protection for members of the community who are living with HIV. In fact, an analysis of community members’ experiences of HIV care revealed a complex reality of illness and knowledge management.

According to the study’s results, although outreach care provided a technical solution for patients living in poorer, remote communities, it also created the need to navigate between care and social realities [48,50]. As the interviewees explained, the complex relationships stemming from the need to “navigate” care extended beyond ART to other social relations and access-related decisions. In their experience, these issues were central to the conflict between the benefits and barriers associated with outreach care.

The paradox of knowledge and stigma, and the conflict between the benefits of outreach care and the difficulties and barriers posed by such care, lie at the heart of the treatment experience for many people on the receiving end of ART [3]. In the case of the current study’s participants, the unintended consequences of outreach care included the need to negotiate treatment and develop coping strategies. These strategies included receiving care at a remote hospital (instead of the local outreach center) or learning to live with “words of ridicule” and “mixed feelings.” The balance between these negotiation strategies was the real-life manifestation of managing illness and treatment experiences in outreach centers in remote rural communities. Although the expansion of ART in rural communities has brought care and relief to many of the world’s poorest people, it has yet to bring chronic normalcy or tranquility to patients’ lives [5,6]. This study underscores the fact that despite acknowledging the benefits of care, many still choose to compromise on privacy or quality of care while navigating complex choices and the implications of those choices. For many, despite access to ART, the battle against AIDS has yet to be won.

The study’s limitations include Zimbabwe’s unique economic and political realities, which are important factors in shaping participants’ lives. The study also focused on ART services provided by a mission hospital, which is the exception rather than the rule in many Southern and Central African Communities. Another important limitation stems from the chosen sample of communities and participants. Although the sample of communities was chosen purposefully to represent the variety of stakeholders participating in HIV care programs, it was by no means a representative sample of said communities. In addition, there was an unavoidable selection bias in terms of participants. To address these limitations, we extended our invitation to participate in the study to various stakeholders in the communities served by the hospital.

The complex negotiations of HIV treatment clearly took their toll on the study participants. An older female interviewee who had been living with HIV for decades talked about how changes in treatment policies had shaped her personal illness experience. Though she acknowledged that HIV outreach clinics had brought a positive change, when asked about stigma she said, “Is there anyone who likes a person who is HIV-positive? No one likes an HIV-positive person.”

In her experience, even if treatment continues to improve and knowledge increases, people with HIV will never be considered normal or loved. The experience of being disliked due to her HIV status further complicated her treatment decisions.

## Conclusions

This study of an outreach model of ART mobile clinics shows that it has simultaneously increased and decreased stigma in the communities in which this model operates. Furthermore, although outreach has made treatment more available, patients have continued to manage their treatment against a backdrop of stigma. In addition, although a greater level of knowledge was evident in these communities, the burden shouldered by people being treated in the outreach clinics excluded full normalization of the HIV illness experience. Consequently, although “Treat All” models and the decentralization of services have saved countless lives, there is still much work to be done in supporting people receiving ART as they navigate their treatment. Such assistance in supporting people and acknowledging the role of stigma and other social stressors in their lives could be provided by ART programs and by community health workers, with greater flexibility in their programming within the communities.

More studies should be conducted to follow the work of ART outreach services, still new in many communities. It would also be helpful to understand the relation between the unique political economy in different countries and the impact of these political economies on ART. However, as decentralizing ART is becoming the norm in many countries worldwide, and the discourse of “chronic normality” gains traction, there is much to be learned in terms of the effect of ART outreach care on patients, healthcare providers, and communities.

### Postscript

The study presented in this article was conducted prior to the COVID-19 pandemic. In February 2020, the authors of this article met to revise the draft. In March 2020, Zimbabwe, like many other countries, closed its borders, and our team members found themselves in lockdown in their respective homes in Zimbabwe and abroad. The pandemic brought new challenges to bear on Zimbabweans and heightened old ones. Economic turmoil and political tensions deepened as the already struggling health system had to cope with new demands. Not surprisingly, the pandemic made the life of people living with HIV in Zimbabwe even more complicated.

During the years of the pandemic, we also lost two members of the research team to COVID-19 and cancer and became, to some extent, paralyzed by these events. In September 2022, some of the team members met in Zimbabwe for a much overdue presentation of our results to colleagues. The loss and devastation incurred by the pandemic were felt in our meeting, and it has become clearer to us that much work is needed to understand the impact of the pandemic on people living with HIV in Zimbabwe. Nevertheless, despite the many changes, the challenges presented in this work are still relevant, as well as the discussion on the way forward toward better, and more just, care.
