# Supplementary material for: Mixed feelings: Unintended consequences of HIV outreach care in Zimbabwe
Source: PLoS One. 2026 Jul 31;21(7):e0354747. doi: 10.1371/journal.pone.0354747 (PMC13426937; doi:10.1371/journal.pone.0354747)
Supplement: Attachment — Submitted filename: Zimbabwe ART PLOSOne Review.docx [file pone.0354747.s001.docx]

## Review of “Mixed Feelings: Unintended Consequences of HIV Outreach Care in Zimbabwe”

The authors study the impact of decentralise ART in Zimbabwe by conducting semi-structured interviews with various stakeholders in a mission hospital in Zimbabwe. They specifically look at the impact of ART on perception and stigma PLHIV.

#### Minor comments

What is meant with “everyday realities of outreach” in the abstract?

Do not use abbreviations that has not been defined, e.g. PLHIV

Better define what is meant with decentralised ART

No citations given for various statements, including (not all listed):

- Studies on HIV care and stigma have found mixed results about the effect of the "treatment for all" policies on the reduction of HIV-related stigma (pg.4)
- Based on a survey conducted in rural Tanzania, Agnarson et al. [YEAR missing] (pg.4)

Pg. 6: “In Mozambique, Kalofanos documented the impact of hunger and conflicts related to food support and ART (43).” Please give more information on the impact, positive or negative, the magnitude, etc.

Pg. 6: “Other studies have shown the unintended consequences of stigma itself, framing it as a pull-push factor to care and not as a barrier” Please clarify the meaning of this statement

Method: give information on how many potential respondents declined to take part after being approached. Also give information on how participants were approached and asked to take part.

The mention of 90-90-90 in the results could be moved to the introduction and needs to be explained.

Please do additional proofreading of the entire paper.

#### Mayor comments

The gap in literature, and contribution of the paper is not clear. This should be very clearly specified in the introduction. In addition, the research question is also stated clearly enough

More information is needed on the composition/description of the respondents, how many were health care providers, community leaders, patients, etc.

Thematic analysis of the interviews would give a better understanding of the overall results
