# Supplementary material for: Mixed feelings: Unintended consequences of HIV outreach care in Zimbabwe
Source: PLoS One. 2026 Jul 31;21(7):e0354747. doi: 10.1371/journal.pone.0354747 (PMC13426937; doi:10.1371/journal.pone.0354747)
Supplement: Attachment — Submitted filename: RESPONSE TO REVIEWER.docx [file pone.0354747.s004.docx]

February 7, 2025

Editorial Board

Plos One

**Reply to Reviewers PONE-D-24-30309 - Mixed Feelings: Unintended Consequences of HIV Outreach Care in Zimbabwe**

Dear editors,

We extend our sincere gratitude to you and the reviewers for the valuable suggestions and constructive critique of the manuscript. The manuscript has been revised to address the editorial and reviewers’ comments. All changes are highlighted at in the text, please see the following responses to the reviewers:

**Reviewer #1:**
**Comment:** proofreading, that includes removing more emotional language, e.g. " Closer to home, no longer secret, and dramatically reducing the physical barriers to care and the long-lasting stigma that resulted from treating only the gravely ill... ", as well as making sure all necessary statements are cited.
**Response**: Thank you for highlighting the issue. The section was revised to avoid overly emotional or jargon specific language. Please see changes made on page 6.

**Comment:** The gap in literature, and contribution of the paper is not clear. This should be very clearly specified in the introduction. In addition, the research question is also stated clearly enough
**Response:** Thank you for highlighting the gaps in the introduction and literature review. Following this comment, the Introduction (page 2) now clarifies the research question and the gap in the literature as well as the argument made in the article. The gaps in the literature were also highlighted in the literature review on pages 4-5

**Comment:** More information is needed on the composition/description of the respondents, how many were health care providers, community leaders, patients, etc.
**Response**: Thank you for this very important note. A table summarizing respondents’ information (Table 1) was added to the article (page 10). A description of participants composition was added to the Results section (page 10). The Methods section was revised to provide more detailed information on study design, recruitment and analysis (page 7-8).

**Comment:** The interviews with 60 participants could have interesting findings, but the current version is not making the argument sufficiently. For example, important information on the method is still missing, and a broader thematic analysis of the interviews could provide more in-depth insights.
**Response:** Thank you for this very important comment. To clarify the presentation of findings in the article, the Methods section was revised to include more information on population, data collection and analysis (pages 7-8). Information of participants was added to the Results section (page 10). In addition, the Results section now includes a detailed description of the analysis process and the analytical framework, as well as the focus of this specific publication (page 11).

**Reviewer #2:**
**Comment:** The manuscript currently lacks demographic information about the study participants (e.g., sex, age, education background)
**Response:** Thank you for this very important note. The Methods section was revised to provide more detailed information on study design, recruitment and analysis (page 7-8). A table summarizing participants’ demographic information (Table 1) was added to the article. A description of participants composition was added to the Results section (page 10).

**Comment:** I recommend adding quantitative analysis where applicable to strengthen the study’s rigor - key themes, frequencies, or demographic breakdowns
**Response**: We are grateful for this suggestion! We went back to our data to produce appropriate figures to demonstrate both the rigor of the analysis and the results. The Methods and Results section were revised to include a summary of participants' demographic information (Table 1 on page 10), as well as figures and explanations of the analysis process including the distribution of codes by frequencies (Figure 1, page 9) framework analysis categories (Figure 2, page 11).

**Comment**: Out of the 60 interviews conducted, only 9 are discussed in the results section. It would be beneficial if the authors could clarify the relevance of these 9 interviews in relation to the entire dataset. A brief explanation of whether these examples are representative, typical, or outlier cases would improve transparency and provide readers with a sense of how these findings apply to the broader study population.
**Response:** Thank you for this very important observation. The Methods and Results sections were revised to offer more information on the process of data analysis(pages 7-11), the characteristics of the participants and the use of quotes as representatives of trends in the data set (pages 8,10). In addition, more supportive quotes, from different interviewees were added to the text in the results section to substantiate the argument (please see addition quotes on pages 1214, 16,17, 19). This elaboration helped us strengthen our argument about the complexity of stigma, and we are grateful to the reviewer for their suggestion on that matter.

**Reviewer #3:**
**Comment**: The gap in literature, and contribution of the paper is not clear. This should be very clearly specified in the introduction. In addition, the research question is also stated clearly enough
**Response:** Thank you for highlighting this gap. The Introduction (page 2) was revised and now clarifies the research question and the gap in the literature as well as the argument made in the article. The gaps in the literature were also highlighted in the literature review on pages 4-5

**Comment**: More information is needed on the composition/description of the respondents, how many were health care providers, community leaders, patients, etc.
**Response:** Thank you for this very important note. A table summarizing participants’ demographic information (Table 1) was added to the article (page 10). A description of participants composition was added to the Results section (page 10). The Methods section was revised to provide more detailed information on study design, recruitment and analysis (page 7-8).

**Comment:** Thematic analysis of the interviews would give a better understanding of the overall results
**Response**: Thank you for this comment. We acknowledge that the description of the analysis process as well as the technics used were not clearly identified in earlier drafts. The methods section was revised to include more information on the processes of analysis, and the themes and categories that were explored in the paper (page 8-9). The Results section was revised to include an introduction of the framework analysis and the categories drawn from the thematic analysis (page 11)

**Minor comments**

**Comment:** What is meant with “everyday realities of outreach” in the abstract?
**Response:** Thank you for this comment. To clarify our intentions the abstract was revised and now reads "the practices and experiences" to avoid overly jargon specific language (page 2).

**Comment:** Do not use abbreviations that has not been defined, e.g. PLHIV
**Response**: Thank you for highlighting our error. The abbreviation was corrected in the text (page 6)

**Comment:** Better define what is meant with decentralized ART
**Response**: Thank you for highlighting the need for further clarification surrounding the practices of decentralization of ART. An explanation of the term decentralization of ART, and its uses in the Zimbabwean context, were added to the text with proper citation (page 6, page 4, page 24, page 25)

**Comment**: No citations given for various statements, including (not all listed):

1. Studies on HIV care and stigma have found mixed results about the effect of the
   "treatment for all" policies on the reduction of HIV-related stigma. Based on a survey conducted in rural Tanzania, Agnarson et al. [YEAR missing]
   (pg.4)
2. Pg. 6: “In Mozambique, Kalofanos documented the impact of hunger and conflicts related to food support and ART (43).” Please give more information on the impact, positive or negative, the magnitude, etc.
3. Pg. 6: “Other studies have shown the unintended consequences of stigma itself, framing it as a pull-push factor to care and not as a barrier” Please clarify the meaning of this statement

   **Response:** Thank you for highlighting the gaps and inconsistencies in the literature review. The following changes were made to the text:
   1. The publication year of the article was added - Angarson et al, 2013 - (p.4)
   2. An explanation regarding the impact of hunger on people living with HIV and their communities was added. Elaborating on the findings of Kalofonos that food donation directed towards only People Living with HIV has also created conflicts and tensions among communities (p.6)
   3. An explanation was added regarding the term "Pull Push Factor" to care in the context of stigma. Elaborating on studies that show how ART can help disclose sickness status, by making the patience un-symptomatic, and therefore helping to avoid stigma. In this way stigma is the pull factor for adherence (p.6)
   4. To address the reviewer comment about missing details in the literature review, the section was revised and with more supporting citations added to two paragraphs: To the section of HIV history in Zimbabwe (p.3), we added source (11)- Garbus L, Khumalo-Sakutukwa G. Country AIDS Policy Analysis Project: HIV/AIDS in Zimbabwe AIDS Policy Research Center, University of California San Francisco 2003.

To the sentence: " The number of centers providing ART increased progressively, from five in June 2004 to 48 in September 2005, covering 32 of 60 districts", we added source (12) - EPFAR Z. The United States President’s Emergency Plan for AIDS Relief (PEPFAR) Zimbabwe Harare, Zimbabwe: PEPFAR; 2015. (page 5).

**Comment**: Method: give information on how many potential respondents declined to take part after being approached. Also give information on how participants were approached and asked to
take part.
**Response:** Thank you for this comment and the invitation to elaborate on the procedural aspects of the study. The invitation to join the study was shared through community health workers, community leaders and health workers. The study team did not approach potential participants directly. An explanation about the recruitment processes was added to the Methods section (page 8). We also added more details to the discussion to address the limitations of the study in terms of participant recruitment, representation and selection bias (page 23).

**Comment:** The mention of 90-90-90 in the results could be moved to the introduction and needs to be explained.
**Response**: We are grateful for this very important comment. The introduction was revised to include an explanation of the 90-90-90 targets with the appropriate reference (page 2).

**Commen**t: Please do additional proofreading of the entire paper.
**Response**: Thank you for highlighting the need for additional proofreading. Additional proofreading of the entire paper was conducted

**General comments:**
**Comment:** Please ensure that your manuscript meets PLOS ONE's style requirements, including those for file naming.
**Response:** Thank you for the comment, PLOS ONE's style requirements, including those for file naming were addressed in the revised version.

**Comment:** Please state what role the funders took in the study. If the funders had no role, please state: "The funders had no role in study design, data collection and analysis, decision to publish, or preparation of the manuscript."
**Response:** The submission materials now include the statement “The funders had no role in study design, data collection and analysis, decision to publish, or preparation of the manuscript."

**Comment:** restriction on data availability : a) If there are ethical or legal restrictions on sharing a de-identified data set, please explain them in detail (e.g., data contain potentially identifying or sensitive patient information, data are owned by a third-party organization, etc.) and who has imposed them (e.g., a Research Ethics Committee or Institutional Review Board, etc.). Please also provide contact information for a data access committee, ethics committee, or other institutional body to which data requests may be sent.
**Response:** The ethical approval granted for the study by the Medical Research Council of Zimbabwe does not include the sharing of the data set. Due to the intimate nature of the interview materials, and the size and structure of the communities and clinics studied, it is impossible to deidentify the transcripts as they include life histories and experiences of respondents that would serve as identifying information.

**Comment**: data availability statement: If you are unable to adhere to our open data policy, please kindly revise your statement to explain your reasoning and we will seek the editor's input on an exemption. Please be assured that, once you have provided your new statement, the assessment of your exemption will not hold up the peer review process.
**Response**: Data statement was revised.

We are grateful to the editors and reviewers for their comments. Attached please find the revised version of the manuscript.
