# Supplementary material for: Mixed feelings: Unintended consequences of HIV outreach care in Zimbabwe
Source: PLoS One. 2026 Jul 31;21(7):e0354747. doi: 10.1371/journal.pone.0354747 (PMC13426937; doi:10.1371/journal.pone.0354747)
Supplement: Attachment — Submitted filename: RESPONSE TO REVIEWER round 2 (1).docx [file pone.0354747.s005.docx]

June , 2025

Editorial Board

Plos One

**Reply to Reviewers (round 2) PONE-D-24-30309R1- Mixed Feelings: Unintended Consequences of HIV Outreach Care in Zimbabwe**

Dear editors,

We extend our sincere gratitude to you and the reviewers for the valuable suggestions and constructive critique of the manuscript. The manuscript has been revised to address the editorial and reviewers’ comments. All changes are highlighted at in the text, please see the following responses to the reviewers:

**Reviewer #2:**
**Comment:** In Table 1 suggests to add participants' ages as a median with a range
**Response**: Thank you for this comment, we have modified Table 1 according to your suggestion and presented the age as a median with range.

**Comment:** The authors organize the analyzed framework into 24 codes, which are further summarized into seven categories. However, no statistical comparison is provided among these codes and categories. Additionally, it is unclear to what extent these codes and categories contribute to the study’s conclusion.

**Response**: Thank you for this very important comment. The use of narrative analysis (Braun and Clarke) was elaborated in the methodology and finding sections. We did not perform as statistical analysis of the codes as if did not play a role in the narrative analysis. Please see a clarification of the process on pages 9 and 23 (with proper citation)

**Reviewer #3:**
**Overall Comments**

**Comment:** Authors should be guided by journal guidelines available: https://journals.plos.org/plosone/s/submission-guidelines
**Response**: Thank you for referring us to the guidelines. The manuscript was amended accordingly.

**Comment:** It provides a lot of detail in the introduction and by the time one gets to the results (perhaps the critical section), one is tired and does not get the key takeaways. For example, introduction is overlong. Introduction should put the study into context. Should state the research question. Should have a clear statement on aims/objectives-why study was conducted and what it aimed to show. Cite a few other studies to put yours into perspective but this section should not be a review of literature. Introduction should be about 1-1.5 pages long.

**Response**: Thank you for this comment. the Introduction section of the article includes the background and literature review relevant to the study. It also include additional information requested by reviewers. We added a sub-heading for background and literature review to clearly delineate the sections (page 3)

**Comment:** some of the detail in the Methods could be left out.

**Response**: Thank you for this comment. The methods section was written to address all aspects of the study and additional information requested in the previous round of review. Therefore, we refrained from making changes at this stage.

**Comment:** As part of this study, we conducted 60 semi-structured interviews with community leaders, healthcare providers, patients, and community members in the areas served by the mission hospital and its outreach clinics. I am assuming “this study” is referring to the CBART study? What was the purpose of conducting the 60 interviews? Was it as part of process evaluation?

**Response**: Thank you for the comment. The relationship between the broader CBART studies and the CBAR ethnographic study were clarified in the methods section (page 8)

**Comment:** Need to be careful with language e.g., infected people, internalized stigma, positive people, HIV positive man

**Response**: Thank you for the comment. In consultation with the study team, and the original language transcripts, the manuscript was edited to reflect these concerns.

**Comment:** It appears that the analysis identified 24 codes (Fig 1) and seven categories, themes I assume. However, results present 3 broad themes – knowledge of ART, persistence of HIV stigma, mixed feelings about outreach care. It is unclear how the main themes came down from seven to three. It may be that the three themes need to have sub-themes?

**Response**: Thank you for this very important comment. The use of narrative analysis (Braun and Clarke) was elaborated in the methodology and finding sections. Please see a clarification of the process on pages 9 and 12 (with proper citation).

**Comment:** Also, did the seven categories necessarily emerge (as per line 309)? If researchers were using a Framework approach, these seven items appear to be issues they would set out to explore from the outset.

**Response** Clarification regarding the analysis were added to the methodology and finding sections (pages 9 and 12).

**Comment:** Related to 3, researchers need to include the interview guide as a supplemental file and refer to it as (S1 File) in text.

**Response**: Thank you for pointing out the missing appendix. Interview guides in Shona and English were submitted as supplemental materials.

**Comment:** Figure 2. It is unclear what the percentages represent especially as it shows seven categories from 24 codes in figure 1. One would think that the 24 codes were later categorized into seven themes

**Response**: Thank you for bringing this unclarity to our attention. Clarification regarding the analysis were added to the methodology and finding sections (pages 9 and 12).

**Abstract**

**Comment:** Should be structured into the following sub-headings (and not exceed 300 words): Background – Should briefly contextualize the study (4-5 sentences)

Methods – Should briefly describe the study including period it was conducted and how data were processed and analyzed.

Results – Could constitute the bulk of the abstract including total number of participants, any key demographic details and then go on to present a fair summary of the results. Currently, just about a sentence - our study reveals that stigma and complex social relations significantly affect the practices and experiences of outreach ART care of both patients and health providers.

Conclusions – Ok as is - The study’s results highlight the need to understand the consequences of the decentralization of HIV care, as well as the barriers and facilitators associated with outreach ART care, to improve the quality of care and provide true accessibility to HIV care for all.

**Response**: Thank you for these helpful comments and the clear guidance on the necessary changes. Abstract was revised per reviewer comments**.**

**Introduction**

**Comment:** Line 83 - decentralization of HIV care (explain what you mean by this).

**Response**: Thank you for the comment. An explanation of the term "decentralization" was added to the introduction with the appropriate reference (page 3) and is later addressed again in the discussion (page 23)

**Comment:** Lines 84- 90 highlighted section should be moved to the Discussion.

**Response**: This section of the introduction presents the main arguments of the article and was elaborated at the request of reviewers in the previous round. Therefore, it was not moved to the Discussion.

**Comment:** Line 122- The number of centers providing ART increased progressively, from five in June 2004 to 48 in September 2005, covering 32 of 60 districts – There are a total of 68 districts in Zimbabwe. What happened to the other 8?

**Response**: Thank you for bringing this to our attention. Upon further confirmation, only 60 districts are addressed in the 2005 report.

**Comment:** Line 138 – Define internal stigma (please note that terminology has moved from internalized to internal stigma) – see Heniff L et al. Reported impact of creativity in the Wakakosha ('You're Worth It') internal stigma intervention for young people living with HIV in Harare, Zimbabwe. PLOS Glob Public Health. 2024 Nov 5;4(11):e0003909. doi: 10.1371/journal.pgph.0003909.

**Response**: Thank you for pointing this out, we have changed the terminology to internal stigma, defined it and added the suggested reference (page 5)

**Comment:** Lines 179-188 could be left out.

**Response**: Thank you for highlighting this issue. In consultation with the study team, we have decided to maintain the original version.

**Methods**

**Comment:** Participants were selected using a purposeful snowball sample – repeating lines 244-245.

**Response**: thank you for highlighting the repetition. We have addressed the issue and deleted that repeating information.

**Results**

**Comment:** Line 402 - People did not understand the disease – Is “disease” referring to AIDS or HIV, a condition – could be an issue of translation here. **\**

**Response**: In consultation with the study team, and the original language transcripts, the manuscript was edited to reflect these concerns.

**Discussion**

**Comment:** line 662 - In Zimbabwe, the implementation of ART started in 2004 when the Zimbabwean government joined the "3 by 5" initiative, launched in 2003 by UNAIDS and WHO (65, 66). Repetitive – this has been mentioned in the Introduction.

**Response**: We have addressed the issue and deleted the repeating information in the discussion since it already appeared in the introduction.

**Comment:** Lines 665-670 – should come earlier in Introduction – describing decentralization.

**Response**: Thank you for this comment. The process of decentralization of ART is now addressed in the introduction (page 3)

**Comment:** Lines 692-704 need references.

**Response**: This paragraph is based on the results of the current study therefore not all of the information requires references; we added some sentences to clarify that. For the parts that did need referencing it was added.

**Comment:** Line 703- For many, despite access to ART, the battle against AIDS has yet to be won. Unsure what this means as ART stops/delays progression to AIDS.

**Response**: Thank you for the comment. The first segment of the sentence addresses the complexity of ART at the heart of this study, and participants documented experiences of care not necessarily resolving all the conflicts associated with living with HIV.

**Minor additional comments**

**Comment:** Antiretroviral therapy should be written consistently throughout, currently Anti-Retroviral Therapy/ antiretroviral therapy

**Response**: This was changed throughout the text to Antiretroviral therapy.

**Acknowledgments**

**Comment:** Check journal requirements but I think all listed by name should have provided consent.

**Response**: Thank you for highlighting the issue. The acknowledgment section was revised.

**References**

**Comment:** References need updating. See for example recent papers on stigma/Stigma in Zimbabwe:

- Ferris France, N. et al. (2025). Interventions to reduce self-stigma among people living with HIV: A systematic review.Stigma and Health. Advance online publication. <https://doi.org/10.1037/sah0000600>
- Rich C et al. Exploring the beliefs, experiences and impacts of HIV-related self-stigma amongst adolescents and young adults living with HIV in Harare, Zimbabwe: A qualitative study. PLoS One. 2022 May 18;17(5):e0268498. doi: 10.1371/journal.pone.0268498.
- Ferris France N et al. Wakakosha "You are Worth it": reported impact of a community-based, peer-led HIV self-stigma intervention to improve self-worth and wellbeing among young people living with HIV in Zimbabwe. Front Public Health. 2023 Jul 28;11:1235150. doi: 10.3389/fpubh.2023.1235150
- Heniff L et al. Reported impact of creativity in the Wakakosha ('You're Worth It') internal stigma intervention for young people living with HIV in Harare, Zimbabwe. PLOS Glob Public Health. 2024 Nov 5;4(11):e0003909. doi: 10.1371/journal.pgph.0003909.

**Response**: Thank you for introducing us to current literature, we have read the article and incorporated them in the introduction (page 5)

We are grateful to the editors and reviewers for their comments. Attached please find the revised version of the manuscript.
