# Supplementary material for: Mixed feelings: Unintended consequences of HIV outreach care in Zimbabwe
Source: PLoS One. 2026 Jul 31;21(7):e0354747. doi: 10.1371/journal.pone.0354747 (PMC13426937; doi:10.1371/journal.pone.0354747)
Supplement: Attachment — Submitted filename: RESPONSE TO REVIEWER ROUND 3.docx [file pone.0354747.s006.docx]

November , 2025

Editorial Board

Plos One

**Reply to Reviewers (round 3) - PONE-D-24-30309R2 - Mixed Feelings: Unintended Consequences of HIV Outreach Care in Zimbabwe**

Dear editors,

We extend our sincere gratitude to you and the reviewers for the valuable suggestions and constructive critique of the manuscript. The manuscript has been revised to address the editorial and reviewers’ comments. All changes are highlighted at in the text, please see the following responses to the reviewers:

**Reviewer #2:**
**Comment:** Line 65–66: The sentence “However, the study shows that but that they also had unintended negative consequences” contains a minor error. Please revise to “However, the study shows that …”
**Response**: This sentence has been revised.

**Comment:** Line 274–276: The phrase “community health workers, community leaders and community health workers” appears to duplicate “community health workers.” Kindly check whether this repetition was unintended.

**Response**: The duplicate word was deleted.

**Comment:** Age of participants: Table 1 presents an age range of 20–80, while the text (line 314) describes participants’ ages as ranging from 18 to the mid-70s. Please clarify this discrepancy.

**Response**: Thank you for that comment, the age range is between 24 and 71, and this was clarified in the text and in TABLE 1.

**Reviewer #3:**
**Comment:** All comments have been addressed. The manuscript needs proofreading especially as PLOS ONE does not copyedit accepted manuscripts, so the language in submitted articles must be clear, correct, and unambiguous. Any typographical or grammatical errors should be corrected at revision.

**Response**: Thank you for that important note, we have done proofreading to the whole article. In addition, it has gone through editing of a professional linguistic editor. We hope now it will be suitable to the standards of PLOS ONE journal All the editing changes are visible in the revised manuscript with track changes.

**Comment:** Line 64 - The study reveals that s treatment
Check this - However, the study shows that but that they also had unintended negative consequences. …physical and social barriers to access ART (full stop missing)
**Response**: This sentence has been revised, and a full stop was added.

**Comment:** Line 102…increase the physical and social barriers to access ART. (check spacing at end of sentence.
**Response**: Extra spacing was deleted.

**Comment:** Line 106 – Could delete the heading ‘Background’.
**Response**: Heading was deleted.

**Comment:** Line 228 - the patience (patients)?
**Response**: Thank you for noting that spelling mistake, it has been corrected to patients.

**Comment:** Fig 1 heading says Ditribution (Distribution)?

**Response**: Thank you for noting that spelling mistake, it has been corrected to Distribution.

We are grateful to the editors and reviewers for their comments. Attached please find the revised version of the manuscript.
