# Supplementary material for: Mixed feelings: Unintended consequences of HIV outreach care in Zimbabwe
Source: PLoS One. 2026 Jul 31;21(7):e0354747. doi: 10.1371/journal.pone.0354747 (PMC13426937; doi:10.1371/journal.pone.0354747)
Supplement: Peer review history

# Peer review: Decision Letter 0

**DOI:** 10.1371/journal.pone.0354747.r001

**Authors:** Richard Makurumidze

26 Nov 2024

PONE-D-24-30309

Mixed Feelings: Unintended Consequences of HIV Outreach Care in Zimbabwe

PLOS ONE

Dear Dr. Gluskinos,

Thank you for submitting your manuscript to PLOS ONE. After careful consideration, we feel that it has merit but does not fully meet PLOS ONE’s publication criteria as it currently stands. Therefore, we invite you to submit a revised version of the manuscript that addresses the points raised during the review process.

Please submit your revised manuscript by Jan 10 2025 11:59PM. If you will need more time than this to complete your revisions, please reply to this message or contact the journal office at plosone@plos.org. When you're ready to submit your revision, log on to https://www.editorialmanager.com/pone/ and select the 'Submissions Needing Revision' folder to locate your manuscript file.

Please include the following items when submitting your revised manuscript:

- A rebuttal letter that responds to each point raised by the academic editor and reviewer(s). You should upload this letter as a separate file labeled 'Response to Reviewers'.
- A marked-up copy of your manuscript that highlights changes made to the original version. You should upload this as a separate file labeled 'Revised Manuscript with Track Changes'.
- An unmarked version of your revised paper without tracked changes. You should upload this as a separate file labeled 'Manuscript'.

If you would like to make changes to your financial disclosure, please include your updated statement in your cover letter. Guidelines for resubmitting your figure files are available below the reviewer comments at the end of this letter.

If applicable, we recommend that you deposit your laboratory protocols in protocols.io to enhance the reproducibility of your results. Protocols.io assigns your protocol its own identifier (DOI) so that it can be cited independently in the future. For instructions see: https://journals.plos.org/plosone/s/submission-guidelines#loc-laboratory-protocols. Additionally, PLOS ONE offers an option for publishing peer-reviewed Lab Protocol articles, which describe protocols hosted on protocols.io. Read more information on sharing protocols at https://plos.org/protocols?utm_medium=editorial-email&utm_source=authorletters&utm_campaign=protocols.

We look forward to receiving your revised manuscript.

Kind regards,

Richard Makurumidze

Academic Editor

PLOS ONE

Journal Requirements:

When submitting your revision, we need you to address these additional requirements.

1. Please ensure that your manuscript meets PLOS ONE's style requirements, including those for file naming. The PLOS ONE style templates can be found at https://journals.plos.org/plosone/s/file?id=wjVg/PLOSOne_formatting_sample_main_body.pdf and https://journals.plos.org/plosone/s/file?id=ba62/PLOSOne_formatting_sample_title_authors_affiliations.pdf

2. Thank you for stating the following financial disclosure: “This work was supported with funding from Gilead Sciences. We thank Dennis Israelski for his support.”

Please state what role the funders took in the study. If the funders had no role, please state: "The funders had no role in study design, data collection and analysis, decision to publish, or preparation of the manuscript." If this statement is not correct you must amend it as needed. Please include this amended Role of Funder statement in your cover letter; we will change the online submission form on your behalf.

3. We note that you have indicated that there are restrictions to data sharing for this study. PLOS only allows data to be available upon request if there are legal or ethical restrictions on sharing data publicly. For more information on unacceptable data access restrictions, please see http://journals.plos.org/plosone/s/data-availability#loc-unacceptable-data-access-restrictions. Before we proceed with your manuscript, please address the following prompts: a) If there are ethical or legal restrictions on sharing a de-identified data set, please explain them in detail (e.g., data contain potentially identifying or sensitive patient information, data are owned by a third-party organization, etc.) and who has imposed them (e.g., a Research Ethics Committee or Institutional Review Board, etc.). Please also provide contact information for a data access committee, ethics committee, or other institutional body to which data requests may be sent. b) If there are no restrictions, please upload the minimal anonymized data set necessary to replicate your study findings to a stable, public repository and provide us with the relevant URLs, DOIs, or accession numbers. For a list of recommended repositories, please see https://journals.plos.org/plosone/s/recommended-repositories. You also have the option of uploading the data as Supporting Information files, but we would recommend depositing data directly to a data repository if possible. We will update your Data Availability statement on your behalf to reflect the information you provide.

4. When completing the data availability statement of the submission form, you indicated that you will make your data available on acceptance. We strongly recommend all authors decide on a data sharing plan before acceptance, as the process can be lengthy and hold up publication timelines. Please note that, though access restrictions are acceptable now, your entire data will need to be made freely accessible if your manuscript is accepted for publication. This policy applies to all data except where public deposition would breach compliance with the protocol approved by your research ethics board. If you are unable to adhere to our open data policy, please kindly revise your statement to explain your reasoning and we will seek the editor's input on an exemption. Please be assured that, once you have provided your new statement, the assessment of your exemption will not hold up the peer review process.

[Note: HTML markup is below. Please do not edit.]

Reviewers' comments:

Reviewer's Responses to Questions

**Comments to the Author**

1. Is the manuscript technically sound, and do the data support the conclusions?

The manuscript must describe a technically sound piece of scientific research with data that supports the conclusions. Experiments must have been conducted rigorously, with appropriate controls, replication, and sample sizes. The conclusions must be drawn appropriately based on the data presented.

Reviewer #1: Partly

Reviewer #2: Partly

**********

2. Has the statistical analysis been performed appropriately and rigorously?

Reviewer #1: N/A

Reviewer #2: No

**********

3. Have the authors made all data underlying the findings in their manuscript fully available?

The PLOS Data policy requires authors to make all data underlying the findings described in their manuscript fully available without restriction, with rare exception (please refer to the Data Availability Statement in the manuscript PDF file). The data should be provided as part of the manuscript or its supporting information, or deposited to a public repository. For example, in addition to summary statistics, the data points behind means, medians and variance measures should be available. If there are restrictions on publicly sharing data—e.g. participant privacy or use of data from a third party—those must be specified.

Reviewer #1: No

Reviewer #2: No

**********

4. Is the manuscript presented in an intelligible fashion and written in standard English?

PLOS ONE does not copyedit accepted manuscripts, so the language in submitted articles must be clear, correct, and unambiguous. Any typographical or grammatical errors should be corrected at revision, so please note any specific errors here.

Reviewer #1: Yes

Reviewer #2: Yes

**********

5. Review Comments to the Author

Please use the space provided to explain your answers to the questions above. You may also include additional comments for the author, including concerns about dual publication, research ethics, or publication ethics. (Please upload your review as an attachment if it exceeds 20,000 characters)

Reviewer #1: The paper need additional proofreading, that includes removing more emotional language, e.g. " Closer to home, no longer secret, and dramatically reducing the physical barriers to care and the long-lasting stigma that resulted from treating only the gravely ill... ", as well as making sure all necessary statements are cited.

The gap in literature, and contribution of the paper is not clear. This should be very clearly specified in the introduction. In addition, the research question is also stated clearly enough

More information is needed on the composition/description of the respondents, how many were health care providers, community leaders, patients, etc.

The interviews with 60 participants could have interesting findings, but the current version is not making the argument sufficiently. For example, important information on the method is still missing, and a broader thematic analysis of the interviews could provide more in depth insights.

Reviewer #2: In the research article “Mixed Feelings: Unintended Consequences of HIV Outreach Care in Zimbabwe” by Noa Gluskinos et al., the authors explore both positive and negative impacts of HIV outreach care in Zimbabwe, particularly regarding stigma. Using qualitative data from 60 interviews conducted between January 13 and March 1, 2019, the study reveals how outreach efforts can inadvertently reinforce stigmatization.

Incorporating the suggestions below would, in my view, greatly enhance the impact of the manuscript. Thank you for considering these recommendations.

1. The manuscript currently lacks demographic information about the study participants (e.g., sex, age, education background), which may influence experiences and perceptions of stigma. Presenting this data is crucial, as certain demographic characteristics may play a significant role in how stigmatization manifests and is perceived. Including a demographic summary would allow readers to better understand the context of these findings and any potential influences on stigma-related experiences.

2. While the authors utilized QDA Miner for descriptive qualitative analysis, I recommend adding quantitative analysis where applicable to strengthen the study’s rigor. The addition of figures or charts to present key themes, frequencies, or demographic breakdowns would enhance the clarity and accessibility of the findings. Visual aids could also provide a clearer picture of how widespread certain themes are across the full set of 60 interviews, offering a more balanced and comprehensive view.

3. Out of the 60 interviews conducted, only 9 are discussed in the results section. It would be beneficial if the authors could clarify the relevance of these 9 interviews in relation to the entire dataset. A brief explanation of whether these examples are representative, typical, or outlier cases would improve transparency and provide readers with a sense of how these findings apply to the broader study population.

**********

6. PLOS authors have the option to publish the peer review history of their article (what does this mean?). If published, this will include your full peer review and any attached files.

If you choose “no”, your identity will remain anonymous but your review may still be made public.

**Do you want your identity to be public for this peer review?**  For information about this choice, including consent withdrawal, please see our Privacy Policy.

Reviewer #1: No

Reviewer #2: No

**********

[NOTE: If reviewer comments were submitted as an attachment file, they will be attached to this email and accessible via the submission site. Please log into your account, locate the manuscript record, and check for the action link "View Attachments". If this link does not appear, there are no attachment files.]

While revising your submission, please upload your figure files to the Preflight Analysis and Conversion Engine (PACE) digital diagnostic tool, https://pacev2.apexcovantage.com/. PACE helps ensure that figures meet PLOS requirements. To use PACE, you must first register as a user. Registration is free. Then, login and navigate to the UPLOAD tab, where you will find detailed instructions on how to use the tool. If you encounter any issues or have any questions when using PACE, please email PLOS at figures@plos.org. Please note that Supporting Information files do not need this step.

Attachment Submitted filename: Zimbabwe ART PLOSOne Review.docx

# Author comment: Author response to Decision Letter 1

**DOI:** 10.1371/journal.pone.0354747.r002

15 Feb 2025

February 7, 2025

Editorial Board

Plos One

Reply to Reviewers PONE-D-24-30309 - Mixed Feelings: Unintended Consequences of HIV Outreach Care in Zimbabwe

Dear editors,

We extend our sincere gratitude to you and the reviewers for the valuable suggestions and constructive critique of the manuscript. The manuscript has been revised to address the editorial and reviewers’ comments. All changes are highlighted at in the text, please see the following responses to the reviewers:

Reviewer #1:

Comment: proofreading, that includes removing more emotional language, e.g. " Closer to home, no longer secret, and dramatically reducing the physical barriers to care and the long-lasting stigma that resulted from treating only the gravely ill... ", as well as making sure all necessary statements are cited.

Response: Thank you for highlighting the issue. The section was revised to avoid overly emotional or jargon specific language. Please see changes made on page 6.

Comment: The gap in literature, and contribution of the paper is not clear. This should be very clearly specified in the introduction. In addition, the research question is also stated clearly enough

Response: Thank you for highlighting the gaps in the introduction and literature review. Following this comment, the Introduction (page 2) now clarifies the research question and the gap in the literature as well as the argument made in the article. The gaps in the literature were also highlighted in the literature review on pages 4-5

Comment: More information is needed on the composition/description of the respondents, how many were health care providers, community leaders, patients, etc.

Response: Thank you for this very important note. A table summarizing respondents’ information (Table 1) was added to the article (page 10). A description of participants composition was added to the Results section (page 10). The Methods section was revised to provide more detailed information on study design, recruitment and analysis (page 7-8).

Comment: The interviews with 60 participants could have interesting findings, but the current version is not making the argument sufficiently. For example, important information on the method is still missing, and a broader thematic analysis of the interviews could provide more in-depth insights.

Response: Thank you for this very important comment. To clarify the presentation of findings in the article, the Methods section was revised to include more information on population, data collection and analysis (pages 7-8). Information of participants was added to the Results section (page 10). In addition, the Results section now includes a detailed description of the analysis process and the analytical framework, as well as the focus of this specific publication (page 11).

Reviewer #2:

Comment: The manuscript currently lacks demographic information about the study participants (e.g., sex, age, education background)

Response: Thank you for this very important note. The Methods section was revised to provide more detailed information on study design, recruitment and analysis (page 7-8). A table summarizing participants’ demographic information (Table 1) was added to the article. A description of participants composition was added to the Results section (page 10).

Comment: I recommend adding quantitative analysis where applicable to strengthen the study’s rigor - key themes, frequencies, or demographic breakdowns

Response: We are grateful for this suggestion! We went back to our data to produce appropriate figures to demonstrate both the rigor of the analysis and the results. The Methods and Results section were revised to include a summary of participants' demographic information (Table 1 on page 10), as well as figures and explanations of the analysis process including the distribution of codes by frequencies (Figure 1, page 9) framework analysis categories (Figure 2, page 11).

Comment: Out of the 60 interviews conducted, only 9 are discussed in the results section. It would be beneficial if the authors could clarify the relevance of these 9 interviews in relation to the entire dataset. A brief explanation of whether these examples are representative, typical, or outlier cases would improve transparency and provide readers with a sense of how these findings apply to the broader study population.

Response: Thank you for this very important observation. The Methods and Results sections were revised to offer more information on the process of data analysis(pages 7-11), the characteristics of the participants and the use of quotes as representatives of trends in the data set (pages 8,10). In addition, more supportive quotes, from different interviewees were added to the text in the results section to substantiate the argument (please see addition quotes on pages 1214, 16,17, 19). This elaboration helped us strengthen our argument about the complexity of stigma, and we are grateful to the reviewer for their suggestion on that matter.

Reviewer #3:

Comment: The gap in literature, and contribution of the paper is not clear. This should be very clearly specified in the introduction. In addition, the research question is also stated clearly enough

Response: Thank you for highlighting this gap. The Introduction (page 2) was revised and now clarifies the research question and the gap in the literature as well as the argument made in the article. The gaps in the literature were also highlighted in the literature review on pages 4-5

Comment: More information is needed on the composition/description of the respondents, how many were health care providers, community leaders, patients, etc.

Response: Thank you for this very important note. A table summarizing participants’ demographic information (Table 1) was added to the article (page 10). A description of participants composition was added to the Results section (page 10). The Methods section was revised to provide more detailed information on study design, recruitment and analysis (page 7-8).

Comment: Thematic analysis of the interviews would give a better understanding of the overall results

Response: Thank you for this comment. We acknowledge that the description of the analysis process as well as the technics used were not clearly identified in earlier drafts. The methods section was revised to include more information on the processes of analysis, and the themes and categories that were explored in the paper (page 8-9). The Results section was revised to include an introduction of the framework analysis and the categories drawn from the thematic analysis (page 11)

Minor comments

Comment: What is meant with “everyday realities of outreach” in the abstract?

Response: Thank you for this comment. To clarify our intentions the abstract was revised and now reads "the practices and experiences" to avoid overly jargon specific language (page 2).

Comment: Do not use abbreviations that has not been defined, e.g. PLHIV

Response: Thank you for highlighting our error. The abbreviation was corrected in the text (page 6)

Comment: Better define what is meant with decentralized ART

Response: Thank you for highlighting the need for further clarification surrounding the practices of decentralization of ART. An explanation of the term decentralization of ART, and its uses in the Zimbabwean context, were added to the text with proper citation (page 6, page 4, page 24, page 25)

Comment: No citations given for various statements, including (not all listed):

1. Studies on HIV care and stigma have found mixed results about the effect of the

"treatment for all" policies on the reduction of HIV-related stigma. Based on a survey conducted in rural Tanzania, Agnarson et al. [YEAR missing]

(pg.4)

2. Pg. 6: “In Mozambique, Kalofanos documented the impact of hunger and conflicts related to food support and ART (43).” Please give more information on the impact, positive or negative, the magnitude, etc.

3. Pg. 6: “Other studies have shown the unintended consequences of stigma itself, framing it as a pull-push factor to care and not as a barrier” Please clarify the meaning of this statement

Response: Thank you for highlighting the gaps and inconsistencies in the literature review. The following changes were made to the text:

1. The publication year of the article was added - Angarson et al, 2013 - (p.4)

2. An explanation regarding the impact of hunger on people living with HIV and their communities was added. Elaborating on the findings of Kalofonos that food donation directed towards only People Living with HIV has also created conflicts and tensions among communities (p.6)

3. An explanation was added regarding the term "Pull Push Factor" to care in the context of stigma. Elaborating on studies that show how ART can help disclose sickness status, by making the patience un-symptomatic, and therefore helping to avoid stigma. In this way stigma is the pull factor for adherence (p.6)

4. To address the reviewer comment about missing details in the literature review, the section was revised and with more supporting citations added to two paragraphs: To the section of HIV history in Zimbabwe (p.3), we added source (11)- Garbus L, Khumalo-Sakutukwa G. Country AIDS Policy Analysis Project: HIV/AIDS in Zimbabwe AIDS Policy Research Center, University of California San Francisco 2003.

To the sentence: " The number of centers providing ART increased progressively, from five in June 2004 to 48 in September 2005, covering 32 of 60 districts", we added source (12) - EPFAR Z. The United States President’s Emergency Plan for AIDS Relief (PEPFAR) Zimbabwe Harare, Zimbabwe: PEPFAR; 2015. (page 5).

Comment: Method: give information on how many potential respondents declined to take part after being approached. Also give information on how participants were approached and asked to

take part.

Response: Thank you for this comment and the invitation to elaborate on the procedural aspects of the study. The invitation to join the study was shared through community health workers, community leaders and health workers. The study team did not approach potential participants directly. An explanation about the recruitment processes was added to the Methods section (page 8). We also added more details to the discussion to address the limitations of the study in terms of participant recruitment, representation and selection bias (page 23).

Comment: The mention of 90-90-90 in the results could be moved to the introduction and needs to be explained.

Response: We are grateful for this very important comment. The introduction was revised to include an explanation of the 90-90-90 targets with the appropriate reference (page 2).

Comment: Please do additional proofreading of the entire paper.

Response: Thank you for highlighting the need for additional proofreading. Additional proofreading of the entire paper was conducted

General comments:

Comment: Please ensure that your manuscript meets PLOS ONE's style requirements, including those for file naming.

Response: Thank you for the comment, PLOS ONE's style requirements, including those for file naming were addressed in the revised version.

Comment: Please state what role the funders took in the study. If the funders had no role, please state: "The funders had no role in study design, data collection and analysis, decision to publish, or preparation of the manuscript."

Response: The submission materials now include the statement “The funders had no role in study design, data collection and analysis, decision to publish, or preparation of the manuscript."

Comment: restriction on data availability : a) If there are ethical or legal restrictions on sharing a de-identified data set, please explain them in detail (e.g., data contain potentially identifying or sensitive patient information, data are owned by a third-party organization, etc.) and who has imposed them (e.g., a Research Ethics Committee or Institutional Review Board, etc.). Please also provide contact information for a data access committee, ethics committee, or other institutional body to which data requests may be sent.

Response: The ethical approval granted for the study by the Medical Research Council of Zimbabwe does not include the sharing of the data set. Due to the intimate nature of the interview materials, and the size and structure of the communities and clinics studied, it is impossible to deidentify the transcripts as they include life histories and experiences of respondents that would serve as identifying information.

Comment: data availability statement: If you are unable to adhere to our open data policy, please kindly revise your statement to explain your reasoning and we will seek the editor's input on an exemption. Please be assured that, once you have provided your new statement, the assessment of your exemption will not hold up the peer review process.

Response: Data statement was revised.

We are grateful to the editors and reviewers for their comments. Attached please find the revised version of the manuscript.

Attachment Submitted filename: RESPONSE TO REVIEWER.docx

# Peer review: Decision Letter 1

**DOI:** 10.1371/journal.pone.0354747.r003

**Authors:** Richard Makurumidze

20 Apr 2025

PONE-D-24-30309R1

Mixed Feelings: Unintended Consequences of HIV Outreach Care in Zimbabwe

PLOS ONE

Dear Dr. Gluskinos,

Thank you for submitting your manuscript to PLOS ONE. After careful consideration, we feel that it has merit but does not fully meet PLOS ONE’s publication criteria as it currently stands. Therefore, we invite you to submit a revised version of the manuscript that addresses the points raised during the review process.

Please submit your revised manuscript by Jun 04 2025 11:59PM. If you will need more time than this to complete your revisions, please reply to this message or contact the journal office at plosone@plos.org. When you're ready to submit your revision, log on to https://www.editorialmanager.com/pone/ and select the 'Submissions Needing Revision' folder to locate your manuscript file.

Please include the following items when submitting your revised manuscript:

- A rebuttal letter that responds to each point raised by the academic editor and reviewer(s). You should upload this letter as a separate file labeled 'Response to Reviewers'.
- A marked-up copy of your manuscript that highlights changes made to the original version. You should upload this as a separate file labeled 'Revised Manuscript with Track Changes'.
- An unmarked version of your revised paper without tracked changes. You should upload this as a separate file labeled 'Manuscript'.

If you would like to make changes to your financial disclosure, please include your updated statement in your cover letter. Guidelines for resubmitting your figure files are available below the reviewer comments at the end of this letter.

If applicable, we recommend that you deposit your laboratory protocols in protocols.io to enhance the reproducibility of your results. Protocols.io assigns your protocol its own identifier (DOI) so that it can be cited independently in the future. For instructions see: https://journals.plos.org/plosone/s/submission-guidelines#loc-laboratory-protocols. Additionally, PLOS ONE offers an option for publishing peer-reviewed Lab Protocol articles, which describe protocols hosted on protocols.io. Read more information on sharing protocols at https://plos.org/protocols?utm_medium=editorial-email&utm_source=authorletters&utm_campaign=protocols.

We look forward to receiving your revised manuscript.

Kind regards,

Richard Makurumidze (MBChB, MPH, PhD)

Academic Editor

PLOS ONE

[Note: HTML markup is below. Please do not edit.]

Reviewers' comments:

Reviewer's Responses to Questions

**Comments to the Author**

1. If the authors have adequately addressed your comments raised in a previous round of review and you feel that this manuscript is now acceptable for publication, you may indicate that here to bypass the “Comments to the Author” section, enter your conflict of interest statement in the “Confidential to Editor” section, and submit your "Accept" recommendation.

Reviewer #2: All comments have been addressed

Reviewer #3: (No Response)

**********

2. Is the manuscript technically sound, and do the data support the conclusions?

The manuscript must describe a technically sound piece of scientific research with data that supports the conclusions. Experiments must have been conducted rigorously, with appropriate controls, replication, and sample sizes. The conclusions must be drawn appropriately based on the data presented.

Reviewer #2: Yes

Reviewer #3: Yes

**********

3. Has the statistical analysis been performed appropriately and rigorously?

Reviewer #2: Yes

Reviewer #3: N/A

**********

4. Have the authors made all data underlying the findings in their manuscript fully available?

The PLOS Data policy requires authors to make all data underlying the findings described in their manuscript fully available without restriction, with rare exception (please refer to the Data Availability Statement in the manuscript PDF file). The data should be provided as part of the manuscript or its supporting information, or deposited to a public repository. For example, in addition to summary statistics, the data points behind means, medians and variance measures should be available. If there are restrictions on publicly sharing data—e.g. participant privacy or use of data from a third party—those must be specified.

Reviewer #2: Yes

Reviewer #3: No

**********

5. Is the manuscript presented in an intelligible fashion and written in standard English?

PLOS ONE does not copyedit accepted manuscripts, so the language in submitted articles must be clear, correct, and unambiguous. Any typographical or grammatical errors should be corrected at revision, so please note any specific errors here.

Reviewer #2: Yes

Reviewer #3: Yes

**********

6. Review Comments to the Author

Please use the space provided to explain your answers to the questions above. You may also include additional comments for the author, including concerns about dual publication, research ethics, or publication ethics. (Please upload your review as an attachment if it exceeds 20,000 characters)

Reviewer #2: a. The authors present the distribution of all age intervals of the interviewed participants, which is acceptable. However, presenting the participants' ages as a median with a range would enhance clarity and improve reader comprehension.

b. The authors organize the analyzed framework into 24 codes, which are further summarized into seven categories. However, no statistical comparison is provided among these codes and categories. Additionally, it is unclear to what extent these codes and categories contribute to the study’s conclusion.

Reviewer #3: Overall Comments

1. This is a potentially interesting paper.

2. Authors should be guided by journal guidelines available: https://journals.plos.org/plosone/s/submission-guidelines

3. It provides a lot of detail in the introduction and by the time one gets to the results (perhaps the critical section), one is tired and does not get the key takeaways. For example, introduction is overlong. Introduction should put the study into context. Should state the research question. Should have a clear statement on aims/objectives-why study was conducted and what it aimed to show. Cite a few other studies to put yours into perspective but this section should not be a review of literature. Introduction should be about 1-1.5 pages long.

4. Also some of the detail in the Methods could be left out.

5. As part of this study, we conducted 60 semi-structured interviews with community leaders, healthcare providers, patients, and community members in the areas served by the mission hospital and its outreach clinics.

I am assuming “this study” is referring to the CBART study? What was the purpose of conducting the 60 interviews? Was it as part of process evaluation?

6. Need to be careful with language e.g., infected people, internalized stigma, positive people, HIV positive man

7. It appears that the analysis identified 24 codes (Fig 1) and seven categories, themes I assume. However, results present 3 broad themes – knowledge of ART, persistence of HIV stigma, mixed feelings about outreach care.

It is unclear how the main themes came down from seven to three. It may be that the three themes need to have sub-themes?

8. Also, did the seven categories necessarily emerge (as per line 309)? If researchers were using a Framework approach, these seven items appear to be issues they would set out to explore from the outset.

9. Related to 3, researchers need to include the interview guide as a supplemental file and refer to it as (S1 File) in text.

10. Figure 2. It is unclear what the percentages represent especially as it shows seven categories from 24 codes in figure 1. One would think that the 24 codes were later categorized into seven themes.

Specific Comments

Abstract

Should be structured into the following sub-headings (and not exceed 300 words):

Background – Should briefly contextualize the study (4-5 sentences)

Methods – Should briefly describe the study including period it was conducted and how data were processed and analyzed.

Results – Could constitute the bulk of the abstract including total number of participants, any key demographic details and then go on to present a fair summary of the results.

Currently, just about a sentence - our study reveals that stigma and complex social relations significantly affect the practices and experiences of outreach ART care of both patients and health providers.

Conclusions – Ok as is - The study’s results highlight the need to understand the consequences of the decentralization of HIV care, as well as the barriers and facilitators associated with outreach ART care, to improve the quality of care and provide true accessibility to HIV care for all.

Introduction

Line 83 - decentralization of HIV care (explain what you mean by this).

Lines 84- 90 highlighted section should be moved to the Discussion.

Line 122- The number of centers providing ART increased progressively, from five in June 2004 to 48 in September 2005, covering 32 of 60 districts – There are a total of 68 districts in Zimbabwe. What happened to the other 8?

Line 138 – Define internal stigma (please note that terminology has moved from internalized to internal stigma) – see Heniff L et al. Reported impact of creativity in the Wakakosha ('You're Worth It') internal stigma intervention for young people living with HIV in Harare, Zimbabwe. PLOS Glob Public Health. 2024 Nov 5;4(11):e0003909. doi: 10.1371/journal.pgph.0003909.

Lines 179-188 could be left out.

Methods

Participants were selected using a purposeful snowball sample – repeating lines 244-245.

Results

Line 402 - People did not understand the disease – Is “disease” referring to AIDS or HIV, a condition – could be an issue of translation here.

Discussion

Line 662 - In Zimbabwe, the implementation of ART started in 2004 when the Zimbabwean government joined the "3 by 5" initiative, launched in 2003 by UNAIDS and WHO (65, 66).

Repetitive – this has been mentioned in the Introduction.

Lines 665-670 – should come earlier in Introduction – describing decentralization.

Lines 692-704 need references.

Line 703- For many, despite access to ART, the battle against AIDS has yet to be won. Unsure what this means as ART stops/delays progression to AIDS.

Minor additional comments

Antiretroviral therapy should be written consistently throughout, currently Anti-Retroviral Therapy/ antiretroviral therapy

Acknowledgments

Check journal requirements but I think all listed by name should have provided consent.

References

References need updating. See for example recent papers on stigma/Stigma in Zimbabwe:

• Ferris France, N. et al. (2025). Interventions to reduce self-stigma among people living with HIV: A systematic review.Stigma and Health. Advance online publication. https://doi.org/10.1037/sah0000600

• Rich C et al. Exploring the beliefs, experiences and impacts of HIV-related self-stigma amongst adolescents and young adults living with HIV in Harare, Zimbabwe: A qualitative study. PLoS One. 2022 May 18;17(5):e0268498. doi: 10.1371/journal.pone.0268498.

• Ferris France N et al. Wakakosha "You are Worth it": reported impact of a community-based, peer-led HIV self-stigma intervention to improve self-worth and wellbeing among young people living with HIV in Zimbabwe. Front Public Health. 2023 Jul 28;11:1235150. doi: 10.3389/fpubh.2023.1235150.

• Heniff L et al. Reported impact of creativity in the Wakakosha ('You're Worth It') internal stigma intervention for young people living with HIV in Harare, Zimbabwe. PLOS Glob Public Health. 2024 Nov 5;4(11):e0003909. doi: 10.1371/journal.pgph.0003909.

**********

7. PLOS authors have the option to publish the peer review history of their article (what does this mean?). If published, this will include your full peer review and any attached files.

If you choose “no”, your identity will remain anonymous but your review may still be made public.

**Do you want your identity to be public for this peer review?**  For information about this choice, including consent withdrawal, please see our Privacy Policy.

Reviewer #2: **Yes:**  Siriwat Akapirat

Reviewer #3: No

**********

[NOTE: If reviewer comments were submitted as an attachment file, they will be attached to this email and accessible via the submission site. Please log into your account, locate the manuscript record, and check for the action link "View Attachments". If this link does not appear, there are no attachment files.]

While revising your submission, please upload your figure files to the Preflight Analysis and Conversion Engine (PACE) digital diagnostic tool, https://pacev2.apexcovantage.com/. PACE helps ensure that figures meet PLOS requirements. To use PACE, you must first register as a user. Registration is free. Then, login and navigate to the UPLOAD tab, where you will find detailed instructions on how to use the tool. If you encounter any issues or have any questions when using PACE, please email PLOS at figures@plos.org. Please note that Supporting Information files do not need this step.

Attachment Submitted filename: renamed_81aa5_with line numbers.docx

# Author comment: Author response to Decision Letter 2

**DOI:** 10.1371/journal.pone.0354747.r004

28 Jun 2025

June , 2025

Editorial Board

Plos One

Reply to Reviewers (round 2) PONE-D-24-30309R1- Mixed Feelings: Unintended Consequences of HIV Outreach Care in Zimbabwe

Dear editors,

We extend our sincere gratitude to you and the reviewers for the valuable suggestions and constructive critique of the manuscript. The manuscript has been revised to address the editorial and reviewers’ comments. All changes are highlighted at in the text, please see the following responses to the reviewers:

Reviewer #2:

Comment: In Table 1 suggests to add participants' ages as a median with a range

Response: Thank you for this comment, we have modified Table 1 according to your suggestion and presented the age as a median with range.

Comment: The authors organize the analyzed framework into 24 codes, which are further summarized into seven categories. However, no statistical comparison is provided among these codes and categories. Additionally, it is unclear to what extent these codes and categories contribute to the study’s conclusion.

Response: Thank you for this very important comment. The use of narrative analysis (Braun and Clarke) was elaborated in the methodology and finding sections. We did not perform as statistical analysis of the codes as if did not play a role in the narrative analysis. Please see a clarification of the process on pages 9 and 23 (with proper citation)

Reviewer #3:

Overall Comments

Comment: Authors should be guided by journal guidelines available: https://journals.plos.org/plosone/s/submission-guidelines

Response: Thank you for referring us to the guidelines. The manuscript was amended accordingly.

Comment: It provides a lot of detail in the introduction and by the time one gets to the results (perhaps the critical section), one is tired and does not get the key takeaways. For example, introduction is overlong. Introduction should put the study into context. Should state the research question. Should have a clear statement on aims/objectives-why study was conducted and what it aimed to show. Cite a few other studies to put yours into perspective but this section should not be a review of literature. Introduction should be about 1-1.5 pages long.

Response: Thank you for this comment. the Introduction section of the article includes the background and literature review relevant to the study. It also include additional information requested by reviewers. We added a sub-heading for background and literature review to clearly delineate the sections (page 3)

Comment: some of the detail in the Methods could be left out.

Response: Thank you for this comment. The methods section was written to address all aspects of the study and additional information requested in the previous round of review. Therefore, we refrained from making changes at this stage.

Comment: As part of this study, we conducted 60 semi-structured interviews with community leaders, healthcare providers, patients, and community members in the areas served by the mission hospital and its outreach clinics. I am assuming “this study” is referring to the CBART study? What was the purpose of conducting the 60 interviews? Was it as part of process evaluation?

Response: Thank you for the comment. The relationship between the broader CBART studies and the CBAR ethnographic study were clarified in the methods section (page 8)

Comment: Need to be careful with language e.g., infected people, internalized stigma, positive people, HIV positive man

Response: Thank you for the comment. In consultation with the study team, and the original language transcripts, the manuscript was edited to reflect these concerns.

Comment: It appears that the analysis identified 24 codes (Fig 1) and seven categories, themes I assume. However, results present 3 broad themes – knowledge of ART, persistence of HIV stigma, mixed feelings about outreach care. It is unclear how the main themes came down from seven to three. It may be that the three themes need to have sub-themes?

Response: Thank you for this very important comment. The use of narrative analysis (Braun and Clarke) was elaborated in the methodology and finding sections. Please see a clarification of the process on pages 9 and 12 (with proper citation).

Comment: Also, did the seven categories necessarily emerge (as per line 309)? If researchers were using a Framework approach, these seven items appear to be issues they would set out to explore from the outset.

Response Clarification regarding the analysis were added to the methodology and finding sections (pages 9 and 12).

Comment: Related to 3, researchers need to include the interview guide as a supplemental file and refer to it as (S1 File) in text.

Response: Thank you for pointing out the missing appendix. Interview guides in Shona and English were submitted as supplemental materials.

Comment: Figure 2. It is unclear what the percentages represent especially as it shows seven categories from 24 codes in figure 1. One would think that the 24 codes were later categorized into seven themes

Response: Thank you for bringing this unclarity to our attention. Clarification regarding the analysis were added to the methodology and finding sections (pages 9 and 12).

Abstract

Comment: Should be structured into the following sub-headings (and not exceed 300 words): Background – Should briefly contextualize the study (4-5 sentences)

Methods – Should briefly describe the study including period it was conducted and how data were processed and analyzed.

Results – Could constitute the bulk of the abstract including total number of participants, any key demographic details and then go on to present a fair summary of the results. Currently, just about a sentence - our study reveals that stigma and complex social relations significantly affect the practices and experiences of outreach ART care of both patients and health providers.

Conclusions – Ok as is - The study’s results highlight the need to understand the consequences of the decentralization of HIV care, as well as the barriers and facilitators associated with outreach ART care, to improve the quality of care and provide true accessibility to HIV care for all.

Response: Thank you for these helpful comments and the clear guidance on the necessary changes. Abstract was revised per reviewer comments.

Introduction

Comment: Line 83 - decentralization of HIV care (explain what you mean by this).

Response: Thank you for the comment. An explanation of the term "decentralization" was added to the introduction with the appropriate reference (page 3) and is later addressed again in the discussion (page 23)

Comment: Lines 84- 90 highlighted section should be moved to the Discussion.

Response: This section of the introduction presents the main arguments of the article and was elaborated at the request of reviewers in the previous round. Therefore, it was not moved to the Discussion.

Comment: Line 122- The number of centers providing ART increased progressively, from five in June 2004 to 48 in September 2005, covering 32 of 60 districts – There are a total of 68 districts in Zimbabwe. What happened to the other 8?

Response: Thank you for bringing this to our attention. Upon further confirmation, only 60 districts are addressed in the 2005 report.

Comment: Line 138 – Define internal stigma (please note that terminology has moved from internalized to internal stigma) – see Heniff L et al. Reported impact of creativity in the Wakakosha ('You're Worth It') internal stigma intervention for young people living with HIV in Harare, Zimbabwe. PLOS Glob Public Health. 2024 Nov 5;4(11):e0003909. doi: 10.1371/journal.pgph.0003909.

Response: Thank you for pointing this out, we have changed the terminology to internal stigma, defined it and added the suggested reference (page 5)

Comment: Lines 179-188 could be left out.

Response: Thank you for highlighting this issue. In consultation with the study team, we have decided to maintain the original version.

Methods

Comment: Participants were selected using a purposeful snowball sample – repeating lines 244-245.

Response: thank you for highlighting the repetition. We have addressed the issue and deleted that repeating information.

Results

Comment: Line 402 - People did not understand the disease – Is “disease” referring to AIDS or HIV, a condition – could be an issue of translation here. \

Response: In consultation with the study team, and the original language transcripts, the manuscript was edited to reflect these concerns.

Discussion

Comment: line 662 - In Zimbabwe, the implementation of ART started in 2004 when the Zimbabwean government joined the "3 by 5" initiative, launched in 2003 by UNAIDS and WHO (65, 66). Repetitive – this has been mentioned in the Introduction.

Response: We have addressed the issue and deleted the repeating information in the discussion since it already appeared in the introduction.

Comment: Lines 665-670 – should come earlier in Introduction – describing decentralization.

Response: Thank you for this comment. The process of decentralization of ART is now addressed in the introduction (page 3)

Comment: Lines 692-704 need references.

Response: This paragraph is based on the results of the current study therefore not all of the information requires references; we added some sentences to clarify that. For the parts that did need referencing it was added.

Comment: Line 703- For many, despite access to ART, the battle against AIDS has yet to be won. Unsure what this means as ART stops/delays progression to AIDS.

Response: Thank you for the comment. The first segment of the sentence addresses the complexity of ART at the heart of this study, and participants documented experiences of care not necessarily resolving all the conflicts associated with living with HIV.

Minor additional comments

Comment: Antiretroviral therapy should be written consistently throughout, currently Anti-Retroviral Therapy/ antiretroviral therapy

Response: This was changed throughout the text to Antiretroviral therapy.

Acknowledgments

Comment: Check journal requirements but I think all listed by name should have provided consent.

Response: Thank you for highlighting the issue. The acknowledgment section was revised.

References

Comment: References need updating. See for example recent papers on stigma/Stigma in Zimbabwe:

• Ferris France, N. et al. (2025). Interventions to reduce self-stigma among people living with HIV: A systematic review.Stigma and Health. Advance online publication. https://doi.org/10.1037/sah0000600

• Rich C et al. Exploring the beliefs, experiences and impacts of HIV-related self-stigma amongst adolescents and young adults living with HIV in Harare, Zimbabwe: A qualitative study. PLoS One. 2022 May 18;17(5):e0268498. doi: 10.1371/journal.pone.0268498.

• Ferris France N et al. Wakakosha "You are Worth it": reported impact of a community-based, peer-led HIV self-stigma intervention to improve self-worth and wellbeing among young people living with HIV in Zimbabwe. Front Public Health. 2023 Jul 28;11:1235150. doi: 10.3389/fpubh.2023.1235150

• Heniff L et al. Reported impact of creativity in the Wakakosha ('You're Worth It') internal stigma intervention for young people living with HIV in Harare, Zimbabwe. PLOS Glob Public Health. 2024 Nov 5;4(11):e0003909. doi: 10.1371/journal.pgph.0003909.

Response: Thank you for introducing us to current literature, we have read the article and incorporated them in the introduction (page 5)

We are grateful to the editors and reviewers for their comments. Attached please find the revised version of the manuscript.

Attachment Submitted filename: RESPONSE TO REVIEWER round 2 (1).docx

# Peer review: Decision Letter 2

**DOI:** 10.1371/journal.pone.0354747.r005

**Authors:** Lara Vojnov

12 Oct 2025

PONE-D-24-30309R2

Mixed Feelings: Unintended Consequences of HIV Outreach Care in Zimbabwe

PLOS ONE

Dear Dr. Gluskinos,

Thank you for submitting your manuscript to PLOS ONE. After careful consideration, we feel that it has merit but does not fully meet PLOS ONE’s publication criteria as it currently stands. Therefore, we invite you to submit a revised version of the manuscript that addresses the points raised during the review process.

Please submit your revised manuscript by Nov 26 2025 11:59PM. If you will need more time than this to complete your revisions, please reply to this message or contact the journal office at plosone@plos.org. When you're ready to submit your revision, log on to https://www.editorialmanager.com/pone/ and select the 'Submissions Needing Revision' folder to locate your manuscript file.

Please include the following items when submitting your revised manuscript:

- A rebuttal letter that responds to each point raised by the academic editor and reviewer(s). You should upload this letter as a separate file labeled 'Response to Reviewers'.
- A marked-up copy of your manuscript that highlights changes made to the original version. You should upload this as a separate file labeled 'Revised Manuscript with Track Changes'.
- An unmarked version of your revised paper without tracked changes. You should upload this as a separate file labeled 'Manuscript'.

-->If you would like to make changes to your financial disclosure, please include your updated statement in your cover letter. Guidelines for resubmitting your figure files are available below the reviewer comments at the end of this letter.

If applicable, we recommend that you deposit your laboratory protocols in protocols.io to enhance the reproducibility of your results. Protocols.io assigns your protocol its own identifier (DOI) so that it can be cited independently in the future. For instructions see: https://journals.plos.org/plosone/s/submission-guidelines#loc-laboratory-protocols. Additionally, PLOS ONE offers an option for publishing peer-reviewed Lab Protocol articles, which describe protocols hosted on protocols.io. Read more information on sharing protocols at https://plos.org/protocols?utm_medium=editorial-email&utm_source=authorletters&utm_campaign=protocols.

We look forward to receiving your revised manuscript.

Kind regards,

Lara Vojnov

Academic Editor

PLOS ONE

Journal Requirements:

If the reviewer comments include a recommendation to cite specific previously published works, please review and evaluate these publications to determine whether they are relevant and should be cited. There is no requirement to cite these works unless the editor has indicated otherwise.

Please review your reference list to ensure that it is complete and correct. If you have cited papers that have been retracted, please include the rationale for doing so in the manuscript text, or remove these references and replace them with relevant current references. Any changes to the reference list should be mentioned in the rebuttal letter that accompanies your revised manuscript. If you need to cite a retracted article, indicate the article’s retracted status in the References list and also include a citation and full reference for the retraction notice.

Reviewers' comments:

Reviewer's Responses to Questions

**Comments to the Author**

1. If the authors have adequately addressed your comments raised in a previous round of review and you feel that this manuscript is now acceptable for publication, you may indicate that here to bypass the “Comments to the Author” section, enter your conflict of interest statement in the “Confidential to Editor” section, and submit your "Accept" recommendation.

Reviewer #2: All comments have been addressed

Reviewer #3: (No Response)

**********

2. Is the manuscript technically sound, and do the data support the conclusions?

The manuscript must describe a technically sound piece of scientific research with data that supports the conclusions. Experiments must have been conducted rigorously, with appropriate controls, replication, and sample sizes. The conclusions must be drawn appropriately based on the data presented.

Reviewer #2: Yes

Reviewer #3: Yes

**********

3. Has the statistical analysis been performed appropriately and rigorously?

Reviewer #2: Yes

Reviewer #3: Yes

**********

4. Have the authors made all data underlying the findings in their manuscript fully available?

The PLOS Data policy requires authors to make all data underlying the findings described in their manuscript fully available without restriction, with rare exception (please refer to the Data Availability Statement in the manuscript PDF file). The data should be provided as part of the manuscript or its supporting information, or deposited to a public repository. For example, in addition to summary statistics, the data points behind means, medians and variance measures should be available. If there are restrictions on publicly sharing data—e.g. participant privacy or use of data from a third party—those must be specified.

Reviewer #2: Yes

Reviewer #3: No

**********

5. Is the manuscript presented in an intelligible fashion and written in standard English?

PLOS ONE does not copyedit accepted manuscripts, so the language in submitted articles must be clear, correct, and unambiguous. Any typographical or grammatical errors should be corrected at revision, so please note any specific errors here.

Reviewer #2: Yes

Reviewer #3: Yes

**********

6. Review Comments to the Author

Please use the space provided to explain your answers to the questions above. You may also include additional comments for the author, including concerns about dual publication, research ethics, or publication ethics. (Please upload your review as an attachment if it exceeds 20,000 characters)

Reviewer #2: a. Line 65–66: The sentence “However, the study shows that but that they also had unintended negative consequences” contains a minor error. Please revise to “However, the study shows that …”

b. Line 274–276: The phrase “community health workers, community leaders and community health workers” appears to duplicate “community health workers.” Kindly check whether this repetition was unintended.

c. Age of participants: Table 1 presents an age range of 20–80, while the text (line 314) describes participants’ ages as ranging from 18 to the mid-70s. Please clarify this discrepancy.

Reviewer #3: All comments have been addressed. The manuscript needs proofreading especially as PLOS ONE does not copyedit accepted manuscripts, so the language in submitted articles must be clear, correct, and unambiguous. Any typographical or grammatical errors should be corrected at revision. See for example:

Line 64 - The study reveals that s treatment

Check this - However, the study shows that but that they also had unintended negative consequences.

…physical and social barriers to access ART (full stop missing)

Line 102…increase the physical and social barriers to access ART . (check spacing at end of sentence.

Line 106 – Could delete the heading ‘Background’.

Line 228 - the patience (patients)?

Fig 1 heading says Ditribution (Distribution)?

**********

7. PLOS authors have the option to publish the peer review history of their article (what does this mean?). If published, this will include your full peer review and any attached files.

If you choose “no”, your identity will remain anonymous but your review may still be made public.

**Do you want your identity to be public for this peer review?**  For information about this choice, including consent withdrawal, please see our Privacy Policy.

Reviewer #2: **Yes:**  Siriwat Akapirat

Reviewer #3: No

**********

[NOTE: If reviewer comments were submitted as an attachment file, they will be attached to this email and accessible via the submission site. Please log into your account, locate the manuscript record, and check for the action link "View Attachments". If this link does not appear, there are no attachment files.]

While revising your submission, please upload your figure files to the Preflight Analysis and Conversion Engine (PACE) digital diagnostic tool, https://pacev2.apexcovantage.com/. PACE helps ensure that figures meet PLOS requirements. To use PACE, you must first register as a user. Registration is free. Then, login and navigate to the UPLOAD tab, where you will find detailed instructions on how to use the tool. If you encounter any issues or have any questions when using PACE, please email PLOS at figures@plos.org. Please note that Supporting Information files do not need this step.

# Author comment: Author response to Decision Letter 3

**DOI:** 10.1371/journal.pone.0354747.r006

29 Jun 2026

November , 2025

Editorial Board

Plos One

Reply to Reviewers (round 3) - PONE-D-24-30309R2 - Mixed Feelings: Unintended Consequences of HIV Outreach Care in Zimbabwe

Dear editors,

We extend our sincere gratitude to you and the reviewers for the valuable suggestions and constructive critique of the manuscript. The manuscript has been revised to address the editorial and reviewers’ comments. All changes are highlighted at in the text, please see the following responses to the reviewers:

Reviewer #2:

Comment: Line 65–66: The sentence “However, the study shows that but that they also had unintended negative consequences” contains a minor error. Please revise to “However, the study shows that …”

Response: This sentence has been revised.

Comment: Line 274–276: The phrase “community health workers, community leaders and community health workers” appears to duplicate “community health workers.” Kindly check whether this repetition was unintended.

Response: The duplicate word was deleted.

Comment: Age of participants: Table 1 presents an age range of 20–80, while the text (line 314) describes participants’ ages as ranging from 18 to the mid-70s. Please clarify this discrepancy.

Response: Thank you for that comment, the age range is between 24 and 71, and this was clarified in the text and in TABLE 1.

Reviewer #3:

Comment: All comments have been addressed. The manuscript needs proofreading especially as PLOS ONE does not copyedit accepted manuscripts, so the language in submitted articles must be clear, correct, and unambiguous. Any typographical or grammatical errors should be corrected at revision.

Response: Thank you for that important note, we have done proofreading to the whole article. In addition, it has gone through editing of a professional linguistic editor. We hope now it will be suitable to the standards of PLOS ONE journal All the editing changes are visible in the revised manuscript with track changes.

Comment: Line 64 - The study reveals that s treatment

Check this - However, the study shows that but that they also had unintended negative consequences. …physical and social barriers to access ART (full stop missing)

Response: This sentence has been revised, and a full stop was added.

Comment: Line 102…increase the physical and social barriers to access ART. (check spacing at end of sentence.

Response: Extra spacing was deleted.

Comment: Line 106 – Could delete the heading ‘Background’.

Response: Heading was deleted.

Comment: Line 228 - the patience (patients)?

Response: Thank you for noting that spelling mistake, it has been corrected to patients.

Comment: Fig 1 heading says Ditribution (Distribution)?

Response: Thank you for noting that spelling mistake, it has been corrected to Distribution.

We are grateful to the editors and reviewers for their comments. Attached please find the revised version of the manuscript.

Attachment Submitted filename: RESPONSE TO REVIEWER ROUND 3.docx

# Editor report: Decision Letter 3

**DOI:** 10.1371/journal.pone.0354747.r007

**Authors:** Lara Vojnov

12 Jul 2026

Mixed Feelings: Unintended Consequences of HIV Outreach Care in Zimbabwe

PONE-D-24-30309R3

Dear Dr. Gluskinos,

We’re pleased to inform you that your manuscript has been judged scientifically suitable for publication and will be formally accepted for publication once it meets all outstanding technical requirements.

Within one week, you’ll receive an e-mail detailing the required amendments. When these have been addressed, you’ll receive a formal acceptance letter and your manuscript will be scheduled for publication.

An invoice will be generated when your article is formally accepted. Please note, if your institution has a publishing partnership with PLOS and your article meets the relevant criteria, all or part of your publication costs will be covered. Please make sure your user information is up-to-date by logging into Editorial Manager at Editorial Manager® and clicking the ‘Update My Information' link at the top of the page. For questions related to billing, please contact billing support.

If your institution or institutions have a press office, please notify them about your upcoming paper to help maximize its impact. If they’ll be preparing press materials, please inform our press team as soon as possible -- no later than 48 hours after receiving the formal acceptance. Your manuscript will remain under strict press embargo until 2 pm Eastern Time on the date of publication. For more information, please contact onepress@plos.org.

Kind regards,

Lara Vojnov

Academic Editor

PLOS One

# Editor report: Acceptance letter

**DOI:** 10.1371/journal.pone.0354747.r008

**Authors:** Lara Vojnov

PONE-D-24-30309R3

PLOS One

Dear Dr. Gluskinos,

I'm pleased to inform you that your manuscript has been deemed suitable for publication in PLOS One. Congratulations! Your manuscript is now being handed over to our production team.

At this stage, our production department will prepare your paper for publication. This includes ensuring the following:

* All references, tables, and figures are properly cited

* All relevant supporting information is included in the manuscript submission,

* There are no issues that prevent the paper from being properly typeset

You will receive further instructions from the production team, including instructions on how to review your proof when it is ready. Please keep in mind that we are working through a large volume of accepted articles, so please give us a few days to review your paper and let you know the next and final steps.

Lastly, if your institution or institutions have a press office, please let them know about your upcoming paper now to help maximize its impact. If they'll be preparing press materials, please inform our press team within the next 48 hours. Your manuscript will remain under strict press embargo until 2 pm Eastern Time on the date of publication. For more information, please contact onepress@plos.org.

You will receive an invoice from PLOS for your publication fee after your manuscript has reached the completed accept phase. If you receive an email requesting payment before acceptance or for any other service, this may be a phishing scheme. Learn how to identify phishing emails and protect your accounts at https://explore.plos.org/phishing.

If we can help with anything else, please email us at customercare@plos.org.

Thank you for submitting your work to PLOS ONE and supporting open access.

Kind regards,

PLOS ONE Editorial Office Staff

on behalf of

Dr. Lara Vojnov

Academic Editor

PLOS One
